# An orally available, brain penetrant, small molecule lowers huntingtin levels by enhancing pseudoexon inclusion

**DOI:** 10.1038/s41467-022-28653-6

**Published:** 2022-03-03

**Authors:** Caroline Gubser Keller, Youngah Shin, Alex Mas Monteys, Nicole Renaud, Martin Beibel, Natalia Teider, Thomas Peters, Thomas Faller, Sophie St-Cyr, Judith Knehr, Guglielmo Roma, Alejandro Reyes, Marc Hild, Dmitriy Lukashev, Diethilde Theil, Natalie Dales, Jang-Ho Cha, Beth Borowsky, Ricardo Dolmetsch, Beverly L. Davidson, Rajeev Sivasankaran

**Affiliations:** 1 Novartis Institutes for Biomedical Research, Basel, Switzerland; 2 Novartis Institutes for Biomedical Research, Cambridge, MA USA; 3 The Raymond G Perelman Center for Cellular and Molecular Therapeutics, The Children’s Hospital of Philadelphia, Philadelphia, PA USA; 4 Department of Pathology and Laboratory Medicine, The Perelman School of Medicine, The University of Pennsylvania, Philadelphia, PA USA; 5 Novartis Pharmaceuticals, East Hanover, NJ USA

**Keywords:** Pharmacodynamics, Huntington's disease

## Abstract

Huntington’s Disease (HD) is a progressive neurodegenerative disorder caused by CAG trinucleotide repeat expansions in exon 1 of the huntingtin (*HTT*) gene. The mutant HTT (mHTT) protein causes neuronal dysfunction, causing progressive motor, cognitive and behavioral abnormalities. Current treatments for HD only alleviate symptoms, but cerebral spinal fluid (CSF) or central nervous system (CNS) delivery of antisense oligonucleotides (ASOs) or virus vectors expressing RNA-induced silencing (RNAi) moieties designed to induce mHTT mRNA lowering have progressed to clinical trials. Here, we present an alternative disease modifying therapy the orally available, brain penetrant small molecule branaplam. By promoting inclusion of a pseudoexon in the primary transcript, branaplam lowers mHTT protein levels in HD patient cells, in an HD mouse model and in blood samples from Spinal Muscular Atrophy (SMA) Type I patients dosed orally for SMA (NCT02268552). Our work paves the way for evaluating branaplam’s utility as an  HD therapy, leveraging small molecule splicing modulators to reduce expression of dominant disease genes by driving pseudoexon inclusion.

## Abstract (web summary)

Huntington’s disease (HD) results from the abnormal expansion of CAG repeats in exon 1 of the HTT gene. Here, the authors show that orally available, brain penetrant molecule branaplam lowers HTT transcript by promoting inclusion of a poison exon or pseudoexon.

## Introduction

HD is an autosomal-dominant progressive neurodegenerative disease that is caused by an expansion in a CAG trinucleotide repeat in the *HTT* gene on chromosome 4^1^. Individuals with one *HTT* allele containing 40 or more CAG repeats are invariably affected by the disease, while individuals with fewer than 36 CAG repeats on both alleles do not manifest the disease. The CAG-repeat expansion results in a mutant huntingtin (mHTT) protein, which is associated with neuronal dysfunction and ultimately neuronal death. The disease is characterized by progressive worsening of motor and cognitive function as well as the appearance of psychiatric symptoms. Atrophy of both white matter and gray matter occur in the disease, starting years before the onset of clinical symptoms. HD also causes systemic issues including metabolic, muscular and cardiac impairments^2,3^. Given the monogenic nature of HD, treatments to reduce the presence of mHTT protein in the brain are expected to slow the progression of the disease and delay the onset of disease symptoms in pre-manifest patients. HTT lowering using ASOs, siRNAs or miRNAs in different mouse models reduces mutant HTT (mHTT) in the brain with significant improvements in motor and behavioral deficits^4–9^. In the clinic, these therapies require either surgical delivery of a viral vector for chronic HTT-transcript lowering by RNAi, or repeated infusions into the CSF by lumbar puncture for ASOs.

NVS-SM1 (LMI070), now called branaplam, is a pyridazine derivative with broad CNS and systemic distribution^10^. It was initially developed as a therapeutic for children with spinal muscular atrophy (SMA) as it modulates splicing of the poorly-spliced survival motor neuron 2 (*SMN2*) gene and restores full-length SMN2 transcript and SMN protein levels to normal^10^. Currently, branaplam is in a phase 1/2 clinical trial in infants with SMA (NCT02268552)^11^ and continues to demonstrate good tolerability and efficacy. Mechanistically, branaplam stabilizes the transient interaction between the SMN2 pre-mRNA and the U1 snRNP complex, boosting SMN2 exon7 inclusion^12^. RNA-seq profiling revealed that a small number of exons, preferentially spliced-in by branaplam, are enriched for a non-canonical nGA 3’-exonic motif^10^. Through a detailed re-analysis of our previously described RNA-seq dataset^10^, we found that in addition to the previously reported splice-in events, branaplam induces other splicing changes. Here we show through a series of in vitro, in vivo, and ex vivo human blood sample analyses, that recognition of the non-canonical nGA 3’-exonic motif by branaplam promotes the inclusion of a pseudoexon in human HTT transcripts, causing downregulation of *HTT* mRNAs and protein.

## Results

### RNA sequencing of cells treated with branaplam reveals the inclusion of a pseudoexon and lowering of *HTT* gene expression

**Fig. 1:**
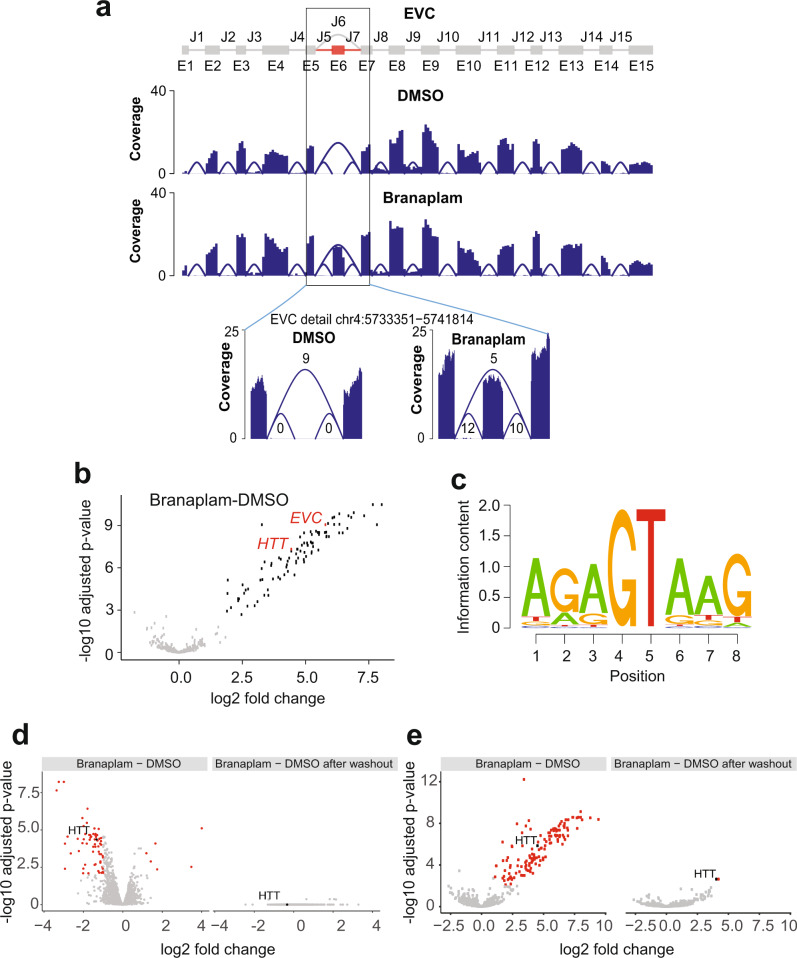
Branaplam promotes pseudoexon definition and inclusion. **a** SGSeq predicted exons for the EVC gene. Pseudoexon E6 is not annotated in UCSC Refseq and is marked in red (top). Coverage plots of the EVC gene for DMSO and branaplam averaged over replicates (introns rescaled) with zoom in on coverage plots for exons E5, E6, and E7, for both DMSO and branaplam are displayed. The average number of junction counts over replicates are shown (bottom). **b** First RNA-seq study. Volcano plot for changes in expression of 577 candidates pseudoexons. The pseudoexon E6 in the EVC gene and the pseudoexon in HTT are marked in red and other pseudoexons with an adjusted *P* value <0.01 and a log_2_ fold change >1 are highlighted in black. Of these 97 candidates, 94 map to GENCODE genes and are shown in Supplementary Table 2. Statistical analysis was performed with limma/voom. *P* values are two-sided and multiplicity adjusted. The adjusted *P* value for the pseudoexon in EVC is 8.86e-10 and the adjusted *P* value for the pseudoexon in *HTT* is 4.82e-08. **c** The panel shows motif enrichment of the 3’ end, in XXX | YYYYY format, (last three bases exonic and first five bases intronic) for the 94 differential pseudoexons that map to GENCODE genes (Supplementary Table 2) obtained with seqLogo. **d** Washout study. Volcano plots for genes differentially regulated after branaplam treatment. The 45 genes highlighted in red are differentially regulated before washout and return to baseline expression 48 h. after washout (left panel). **e** Volcano plot for 1543 pseudoexon candidates before (left) and after washout (right). The pseudoexon in HTT is marked in black. Highlighted in red are 139 candidates before washout and 2 candidates (*HTT* and *TBCA*) after washout with an adjusted *P* value <0.01 and a log_2_ fold change >1. Of these (including HTT), 138 (before washout) and 2 (after washout) respectively map to GENCODE genes and are shown in Supplementary Table 4. Statistical analysis was performed with limma/voom. *P* values are two-sided and multiplicity adjusted. The adjusted *P* value for the pseudoexon in HTT is 1.41e-06 before washout and 2.30e-03 after washout. The adjusted *P* value for the pseudoexon in TBCA after washout is also 2.30e-03.

To investigate further transcripts targeted by branaplam we treated SH-SY5Y human neuroblastoma cells with 100 nM branaplam (5× higher dose than the EC50 for SMN agonism in vitro) for 24 h and confirmed transcript changes using RNA-seq (hereafter referred to as the first RNA-seq study). Like our previous results in Type I SMA fibroblasts, branaplam induced few genome-wide expression changes. 45 genes exhibit an absolute fold change > ±2 with a multiple testing correction (MTC)-adjusted *P* value < 0.01 (Supplementary Table 1). To ascertain exonic changes, reads were aligned to the genome using the STAR aligner^13^. Intron predictions from the gapped alignments show that branaplam promotes the splicing-in of previously unannotated pseudoexons between annotated exons, as exemplified for the Ellis-van Creveld (EVC) gene (Fig. 1a). Here, we use the term of “pseudoexon”(as defined by Sun and Chasin)^14^ to indicate a potential exon, containing adequate 5′ and 3′ splice sites, that is not normally spliced into mature mRNA by the cellular splicing machinery. Genome-wide, 100 nM branaplam promotes the inclusion of 94 unannotated pseudoexons that map to GENCODE genes and show a fold change >2 with an MTC-adjusted *P* value < 0.01 (Fig. 1b and Supplementary Table 2). Like annotated exons spliced-in after branaplam treatment^10,15^, these pseudoexons are highly enriched in an nGA motif at their 3’-end (Fig. 1c and Supplementary Fig. 1a), and in the majority of cases are predicted to introduce a frameshift and/or stop codons.

Since branaplam is dosed once weekly in SMA patients, we mimicked this dosing paradigm in vitro by running a compound washout experiment to assess the reversibility of events triggered by branaplam. In this second RNA-seq experiment (hereafter referred to as washout study) we treated SH-SY5Y cells with 100 nM branaplam for 24 h, washed out the treatment, and carried out RNA-seq after a further 48 h of incubation in cell-culture medium. A full day (24 h) after treatment, 69 genes exhibited an absolute fold change > ±2 with an MTC-adjusted *P* value < 0.01 (Supplementary Table 3). Overall, the results from this study were highly consistent with the first RNA-seq study with minor differences (in this second-reversibility study, 87% of the genes differentially expressed after branaplam treatment behaved similarly to the first study). Forty-eight hours after washout, all 69 genes returned to baseline levels (Fig. 1d, left panel). Similarly, we identified 138 unannotated pseudoexons that map to GENCODE genes and show a fold change >2 with an MTC-adjusted *P* value < 0.01 after branaplam treatment (with 88% of the pseudoexons identified to be differentially expressed in this study and differently expressed in the first RNA-seq study). Forty-eight hours after washout, 136 of the 138 pseudoexons returned to baseline levels (Supplementary Table 4 and Fig. 1e). The pseudoexons in the *HTT* gene and the Tubulin Folding Cofactor A (*TBCA*) gene were the only events that remained significant after washout (Supplementary Table 4). These results highlight the transient and reversible effects of branaplam on nearly all gene and pseudoexon level changes.

**Fig. 2:**
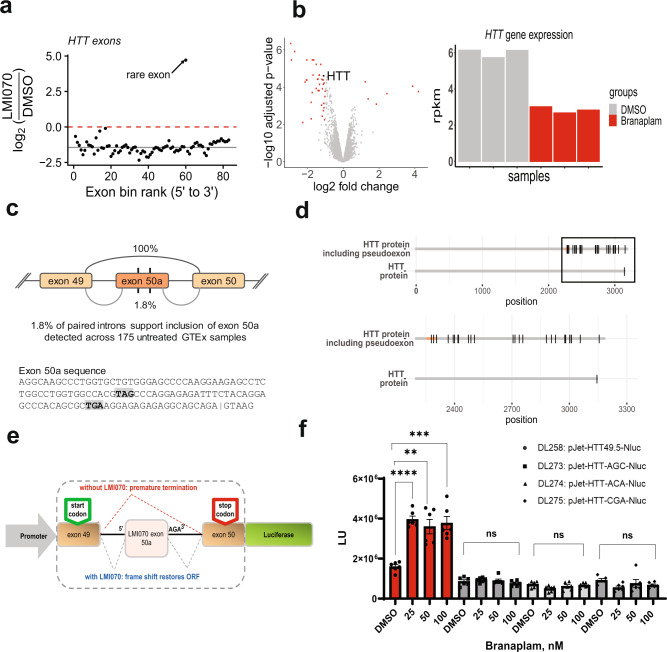
Branaplam enhances inclusion of a pseudoexon in human *HTT*. **a** Washout study. Identification of the novel pseudoexon in *HTT* following branaplam treatment. The *y* axis shows the log_2_ fold change as a function of the exon position rank of the disjoint exon model (from 5’ to 3’). The dark horizontal solid line shows the median of the log_2_ fold changes of the HTT exon and the horizontal red line highlights the zero line of the *y* axis (i.e., no change). All exons are consistently downregulated in branaplam-treated cells with the exception of the novel HTT pseudoexon, which is induced by branaplam. **b** First RNA-seq study. Volcano plot for changes in gene expression in human neuroblastoma cells following treatment with branaplam. Genes with an adjusted *P* value <0.01 and an absolute log_2_ fold change >1 are highlighted in red (left panel). Statistical analysis was performed with limma/voom. *P* values are two-sided and multiplicity adjusted (Benjamini–Hochberg false discovery rate). The adjusted *P* value for the HTT gene is 1.90E-05. The right panel shows human Huntingtin gene expression values from RNA-seq experiments in a SH-SY5Y cells following DMSO (gray) or branaplam (red) treatment. **c** The top panel shows that the *HTT* pseudoexon is a rare exon, detectable in normal human tissue at low levels. The bottom panel shows the sequence of the *HTT* pseudoexon that is spliced-in after treatment with branaplam and induces a frameshift resulting in two stop codons, both in the pseudoexon itself (highlighted by a gray box). **d** The top panel shows the wildtype HTT protein and the HTT protein with the inclusion of the pseudoexon (shown in orange), with black vertical lines depicting the stop codons. The bottom panel is focused on the C-terminal end of both isoforms. Shown as vertical black lines are stop codons that are included when the pseudoexon is induced. **e** Graphic depicting the design of the *HTT* minigene reporters. **f** Data showing the effect of DMSO or branaplam on wild-type and point-mutant *HTT* minigene reporters as measured by luciferase reporter activity. Data represent mean + /−STDEV (*n* = 6). Response of the wildtype reporter (DL258) to indicated doses of branaplam, **P < 0.01, ***P < .001, ****P < 0.0001, Unpaired *t* test with Welch’s correction. Response of the three mutant reporters (DL273, DL274, DL275) to branaplam was not significantly different compared to DMSO. Unpaired *t* test with Welch’s correction.

Among the splice-in events triggered by branaplam treatment was a pseudoexon within the *HTT* gene between exons 49 and 50 (Fig. 2a). Intriguingly, in addition to the spliced-in pseudoexon, *HTT* was also one of the genes downregulated by branaplam treatment compared with the DMSO control (Fig. 2b). We used Snaptron, a resource containing a list of all exon-exon junctions found in 75,806 human and mouse RNA-Seq samples^16^, to assess if this candidate pseudoexon was exclusive to our dataset. We discovered that the unannotated splice-in event between exons 49 and 50 (exon 50a, Snaptron^17^, Fig. 2c) of the *HTT* transcript occurs naturally, albeit at a very low rate of 2% (Fig. 2c). Interestingly, pseudoexon 50a induces a frameshift resulting in several stop codons both in the pseudoexon itself as well as in downstream exons, (Fig. 2c, d), explaining the observed reduction in *HTT* gene expression. The nGA motif at the 3’-exonic end of pseudoexon 50a in the *HTT* gene is poorly conserved across species as shown by an alignment of genomic sequences of the 3’-end of *HTT* gene exon 50a for human, chimpanzee, dog, mouse, and rat, the nGA motif being present only in human and chimpanzee (Supplementary Fig. 1b). Consistent with the absence of the nGA motif at the 3’-end of pseudoexon 50a in mouse, no Htt-transcript downregulation was evident in a murine cell line treated with branaplam nor was a pseudoexon detected (Supplementary Fig. 1c). Together with the results from Palacino et al.^10^, this result supports a key role for the non-canonical nGA 3’-motif in the mode of action of branaplam. We next examined intra-human variations in the *HTT* locus using gnomAD versions 2 and 3^18^. Focusing specifically on the compound-sensitive region proximal to the nGA motif of the pseudoexon, a single, very rare SNP (rs148430407, allele frequencies from 0.00255713 to 0.00269069) was identified in the exon-intron region. These results indicate that the mechanism described herein has the potential to work in most HD patients.

### Branaplam promotes inclusion of pseudoexon 50a  and lowers HTT mRNA and protein levels in vitro

To confirm that *HTT* downregulation was dependent on the 3’-exonic motif, we cloned the DNA sequence spanning human *HTT* exon 49 to exon 50 into a luciferase reporter plasmid. As described in “Methods”, two stop codons in the LMI070-dependent pseudoexon were mutated to maintain an open-reading frame. The resulting *HTT* minigene reporters carry either the wild-type or a mutated version of the nGA 3’exonic motif within exon 50a and were designed to indicate *HTT* exon 50a inclusion based on in-frame luciferase expression (Fig. 2e). While branaplam promoted a robust, dose-dependent increase in activity of the wildtype reporter, this effect was markedly reduced in the context of the nGA point-mutant reporters (Fig. 2f). These results indicate that the nGA motif is required for branaplam-dependent *HTT* downregulation.

**Fig. 3:**
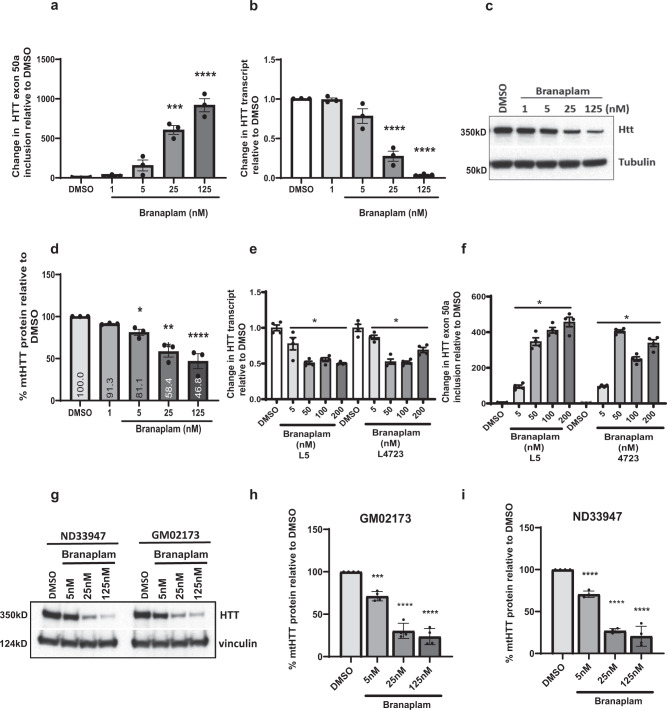
Branaplam lowers HTT-transcript and protein levels in normal and HD patient cells. **a** Quantitative PCR results showing the transcript levels of HTT exon 50a included after DMSO or branaplam treatment (at indicated doses), ****P < 0.0001, one-way ANOVA. The data are presented as mean ± SEM of three independent experiments. **b** HTT-transcript changes after DMSO or branaplam treatment (at indicated doses) for 24 h in SH-SY5Y human neuroblastoma cells, ***P < 0.001, ****P < 0.0001, one-way ANOVA. The data are presented as mean ± SEM of three independent experiments. **c** Western blot and **d** quantitation showing total HTT protein levels after DMSO or branaplam treatment (at indicated doses) for 48 h in SH-SY5Y cells, *P < 0.05, ***P < 0.001, ****P < 0.0001, one-way ANOVA. The data are presented as mean ± SEM of three independent experiments. **e** Quantitative PCR results showing *HTT* mRNA expression levels after DMSO or Branaplam treatment (at indicated doses) for 24 h. Two independent HD patient fibroblasts cell lines (GM04723 and ND31551 (L5)), and a TaqMan™ assay priming for human *HTT* exons 64–65 was used. Samples are normalized to human GAPDH, and data are the mean ± SEM relative to HD fibroblasts treated with DMSO (eight biological replicates, *P value <0.001, one-way ANOVA followed by a Bonferroni’s post hoc). **f** Quantitative PCR results showing HTT50a exon inclusion levels in two HD patient fibroblasts cell lines (GM04723 and ND31551 (L5)) after 24-h treatment with DMSO or branaplam (at indicated doses). Samples are normalized to human GAPDH, and data are the mean ± SEM relative to HD fibroblasts treated with DMSO (four biological replicates, *P value <0.001, one-way ANOVA followed by a Bonferroni’s post hoc). **g** western blot and **h**, **i** quantitation showing mutant HTT protein levels after DMSO or branaplam treatment (at indicated doses) for 96 h in HD patient fibroblast cells, ****P < 0.0001, one-way ANOVA. Data are the mean ± SD relative to DMSO from four independent experiments.

Next, we evaluated branaplam in SH-SY5Y cells and found dose-dependent increases in exon 50a inclusion (Fig. 3a), with a concomitant, dose-dependent decrease of HTT-transcript levels to 30–95% of normal endogenous level (Fig. 3b). Furthermore, western blot analysis revealed that transcript lowering was accompanied by up to 55% reduction of normal HTT protein (Fig. 3c) with the IC50 for HTT lowering being consistent with that previously observed for SMN agonism at approximately 10 nM. To confirm these effects with mHTT protein, we evaluated branaplam in two independent fibroblast lines from HD patients. In both cell lines, there was upregulation of the exon 50a-containing transcript (Fig. 3g), resulting in mHTT downregulation. This effect was assessed using primers upstream (exons 64–65, Fig. 3e) or downstream (exons 1–2, Supplementary Fig. 1d) of the exon 49–50 region. Western blot analysis of lysates from two HD patient fibroblast lines showed dose-dependent lowering of mHTT protein to 70% of normal levels after 96 h of compound treatment (Fig. 3g). While full-length HTT protein is clearly lowered, it is possible that fragments in which the downstream stop codons are operative (and the transcript escapes nonsense-mediated decay) could still be present. We found no evidence of any anomalous truncated fragments in vitro and in subsequent in vivo studies (all western gel images included as supplemental data). Thus, branaplam downregulates normal and *mHTT* gene expression by promoting the inclusion of a pseudoexon that carries in-frame stop codons (Fig. 2d). Several recent studies highlight the occurrence of pseudoexons in disease pathology^19–21^; however, the current work represents an example of a chemically induced pseudoexon for the robust downregulation of a disease-carrying gene.

### Branaplam promotes inclusion of pseudoexon 50a and lowers HTT mRNA and protein levels in vivo after oral dosing in BacHD mice

**Fig. 4:**
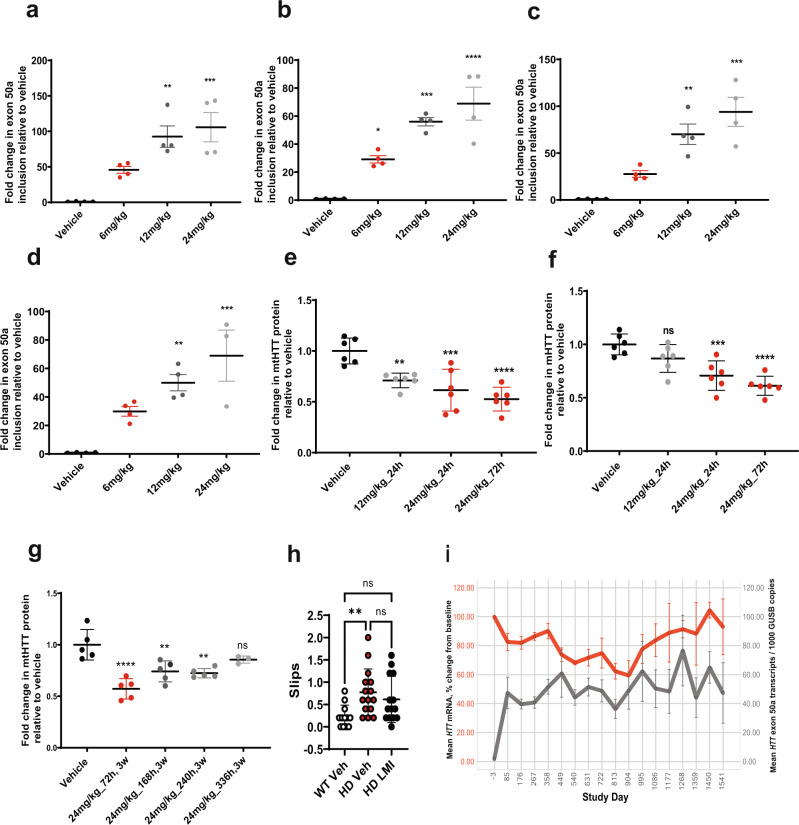
Branaplam modulates human mHTT transcript and protein levels in the brain and rescues narrow beam performance in BacHD mice. Q-PCR analysis of *HTT* Exon 50a inclusion in **a**, striatum **b**, cortex **c**, thalamus and **d**, cerebellum in response to vehicle or branaplam administrated at 6, 12, and 24 mg/kg every other day for a total of three doses. Data are the mean ± SEM of four mice per group, ****P < 0.0001, ***P < 0.001, **P < 0.01, *P < 0.05 vehicle vs branaplam-treated groups, one-way ANOVA followed by Bonferroni’s post hoc. **e** mHTT protein levels in the striatum of BacHD mice (*n* = 6) treated with 12 mg/kg or 24 mg/kg of branaplam for 3 weeks and analyzed at the indicated timepoints after last treatment. Data are the mean ± SEM of six mice per group. One-way ANOVA with Dunnett’s multiple comparisons test: **P = 0.048, ***P = 0.0003, ****P < 0.0001, and (**b**) ****P < 0.0001, **P value = 0.003. **f** mHTT protein levels in the cortex of BacHD mice (*n* = 6) treated with 12 mg/kg or 24 mg/kg of branaplam and taken down at indicated timepoints. Data are the mean ± SEM of six mice per group. One-way ANOVA with Dunnett’s multiple comparisons test: ***P value = 0.0008, (Vehicle vs 24 mg/kg, 72 h) ****P value <0.0001. **g** Time-course of mHTT protein levels in the striatum of BacHD mice (*n* = 6) treated with 12 mg/kg or 24 mg/kg of branaplam for 3 weeks and analyzed at the indicated timepoints after last treatment. Data are the mean ± SEM of six mice per group. One-way ANOVA with Dunnett’s multiple comparisons test: **P value = 0.0017, ****P value <0.0001. **h** Branaplam treatment in BACHD females rescues the number of slips on the narrow beam. Data represent mean ± SEM of 12 WT and 15 BACHD mice treated with vehicle, and 14 HD mice treated with branaplam. *P ≤0.05 and **P ≤0.01 indicates a significant difference detected by a Kruskal–Wallis test with Dunn’s multiple comparisons tests. **i** *HTT* transcripts in whole-blood samples from Type I SMA patients dosed weekly with branaplam. The mean age at screening was 4.21 months (SD, 1.481 months; range, 1.8‒7.5 months) with slightly more females (57.9%) enrolled into the study. The gray curve depicts average number of copies of HTT mRNA transcripts with exon 50a inclusion per 1000 GUSB mRNA copies in the blood of infants with SMA Type I after multiple weekly administrations branaplam. The red curve represents the average percentage change from baseline in total blood HTT mRNA levels in infants with SMA type I after multiple weekly administration of oral branaplam as measured using primers upstream (exons 36–37) of exon 50a. Error Bars represent mean ± SEM.

The BacHD murine model^22^, which harbors the full-length human *mHTT* gene with 97 glutamine repeats, was used for in vivo assessment of mHTT reduction by branaplam. BacHD mice were administered a single oral dose of branaplam at 10 mg/kg or 50 mg/kg, and exon 50a inclusion and *mHTT* mRNA levels were assessed. Quantitative PCR showed a dose-dependent increase in exon 50a inclusion in the brain at 8- and 24 h after dosing with a trend toward returning to baseline levels at the 48-h timepoint in the 50 mg/kg group (Supplementary Fig. 2a). Total *mHTT* transcript levels at both doses showed a trend of decreasing concentrations at 8 h (not significant). At 24 h, in the 50 mg/kg group, a 60–70% reduction of transcript levels was observed relative to the vehicle control (Supplementary Fig. 2b). Similar changes in total *mHTT* and exon 50a-containing *mHTT* transcripts were also seen in peripheral blood samples (Supplementary Fig. 2c, d). To determine if the effect on *HTT* modulation was consistent across various brain regions, BacHD mice were administered three doses of branaplam each at 6, 12, and 24 mg/kg dose levels, and exon 50a inclusion levels were measured. Although three doses of branaplam were insufficient to induce uniform HTT lowering across the brain, there was robust dose-dependent induction of exon 50a inclusion in the cortex, striatum, thalamus, and cerebellum (Fig. 4a–d) demonstrating brain-wide modulation of HTT splicing after oral dosing of branaplam.

To evaluate the effect of branaplam on mHTT protein, BacHD mice were given thrice weekly doses of branaplam at 12 mg/kg or 24 mg/kg for either 1 or 3 weeks. At the end of the 1-week or 3-week treatment, the mice were euthanized at different timepoints, and the tissues were harvested for analysis. At 24 h after the last dose, dose-dependent mHTT protein reduction (up to 40–45%) was observed in the striatum and cortex, relative to BacHD mice treated with the vehicle control (Fig. 4e, f). A trend toward further mHTT protein reduction was apparent 72 h after the last dose (Fig. 4e, f). Comparable mHTT protein lowering in the striatum and cortex was observed in an independent study in which BacHD mice received 16 doses of 24 mg/kg branaplam (Supplementary Fig. 4a, b). The level of mHTT protein reduction observed in the CNS is in line with levels predicted to provide therapeutic benefit based on preclinical observations with other HTT protein lowering modalities^4–6,8,9^. Furthermore, robust lowering of human *HTT* transcript in the blood and mHTT protein in the liver were evident (Supplementary Fig. 3a, b). Branaplam concentrations were determined in the cerebellum (surrogate for overall brain), liver, and muscle. The concentration ratio of branaplam between the cerebellum and plasma was about 1.5, indicating clear distribution to the brain. In the liver and muscle, branaplam concentrations in adult BacHD mice were ~700-fold and 22-fold higher respectively than in the cerebellum. This is in line with the more pronounced mHTT protein reduction observed in peripheral tissues. As noted before in our in vitro studies, the effect of branaplam was specific to human HTT protein and did not impact mouse HTT levels (Supplementary Fig. 3c). Since the sequence motif targeted by branaplam in the *HTT* gene is present only in human and chimpanzee the effects observed in mice cannot readily be demonstrated in larger preclinical animal species. However, the distribution of branaplam into cortex and relevant deep brain regions was shown in dog and NHP confirming broad distribution to key brain regions impacted in HD, such as the caudate and putamen (Supplementary Table 5). The plasma concentrations at 24 h after dosing were up to the Cmax values in Type 1 SMA patients at the highest branaplam dose tested in NHP and somewhat lower in dogs. The observed concentration ratios of branaplam between brain regions and plasma were >fourfold higher than those of mice suggesting a more pronounced distribution of branaplam into the brain of dog and NHP. These data give credence to our therapeutic hypothesis that branaplam will broadly lower HTT protein levels in the cortex and deep brain regions critical to HD pathogenesis following oral dosing in HD patients.

To further elaborate the kinetics of mHTT protein reduction and recovery following branaplam treatment, we carried out a time-course study in which BacHD mice were given thrice weekly doses of branaplam (24 mg/kg) for 3 weeks. The results showed significant mHTT reduction at 72 h, and a return to baseline levels between 72 and 336 h in the striatum (Fig. 4g) and between 72 and 168 h in the cortex (Supplementary Fig. 3d).

The mechanism of action of branaplam relies on the nGA sequence motif which is not conserved between the human and mouse Huntingtin genes. This restricts in vivo evaluation with branaplam to one of two available, full-length human *HTT* transgenic models, i.e., the YAC128 or BacHD mouse models. While motor abnormalities in the YAC128 and the BacHD mouse models are widely reported in published literature, the significant body weight gain seen in these models can confound interpretation of improvements in motor performance. Our unpublished work has identified the narrow beam test as a sensitive assay for evaluating motor performance in BacHD mice without a major influence from body weight changes. To evaluate potential functional rescue with branaplam, 2-month-old BacHD females were treated intermittently with branaplam for 3 months (1st and 2nd month: three doses per week for a total of 12 doses, 3rd month: every other day for a total of 16 doses), and tested on the narrow beam. The narrow beam consists of crossing an elevated beam that progressively narrows to reach a safe platform^23^. This task reveals subtle motor deficits in motor coordination, gait and balance in a quantitative and sensitive manner^24^. Previous reports show that BacHD mice slip more frequently starting early in the disease^25^. We chose females as they show greater motor performance impairments during the age interval at which branaplam treatment took place. Further, we selected a motor test that allowed rapid testing, with a minimal impact of the increased weight on the performance^26^. branaplam-treated BacHD females slip on the narrow beam at a frequency similar to vehicle-treated WT females, unlike vehicle-treated BacHD females, which slip significantly more (*KWS* = 10.26, *N*_*WT-Veh*_ = 12, *N*_*HD-Veh*_ = 15, *N*_*HD-LMI*_ = 14, *P* = 0.006; WT-Veh vs HD-Veh: *Z* = 3.159; *P* = 0.005, *d* = 1.3; Fig. 4h). This provides early evidence that short, intermittent branaplam treatment improves an important measure of motor performance in BacHD mice to WT levels.

### Blood samples from SMA Type I patients treated with branaplam show an increase in exon 50a inclusion and lowering of HTT mRNA

Next, we explored the relationship between branaplam and HTT-transcript levels in humans by assessing whole-blood samples from SMA Type I infants enrolled in an open-label, proof-of-concept study of oral branaplam. These patients received once-weekly doses of branaplam for multiple weeks. Longitudinal gene expression analysis of blood samples from these patients demonstrated exon 50a inclusion after weekly doses of branaplam, with a sustained reduction of HTT-transcript concentrations over 750 study days (Fig. 4i). The inclusion of pseudoexon 50a by branaplam decreased blood *HTT* mRNA levels to ~40% of baseline over a period of 904 study days (Fig. 4i), demonstrating a sustained pharmacodynamic effect and promising initial validation of the translatability of the pseudoexon-driven mechanism of HTT lowering in humans.

## Discussion

Our work describes an example of a small molecule that modulates HTT-transcript splicing to downregulate HTT and mHTT protein levels. The oral availability of branaplam offers a distinct delivery advantage over virally delivered RNAi or repeated intrathecal ASO infusions which provide longer-lasting and more localized lowering that, to date, cannot be rapidly reversed, and may be confounded by ASO toxicities. Moreover, the broad distribution of branaplam in the CNS enables HTT protein reduction throughout the brain, including the cortex and striatum, key brain regions in the neuropathology of HD, as well as any impacted peripheral tissues. This peripheral distribution has the potential to additionally address systemic issues associated with HD. While branaplam modulates splicing of additional genes, the majority of these changes are transient as evidenced by the in vitro compound washout data presented. Importantly, branaplam shows a positive benefit:risk profile in young children with Type 1 SMA, and future clinical trials will evaluate the therapeutic potential for branaplam to slow disease progression in people with HD. While complete removal of normal HTT protein has been shown to have adverse consequences in some animal studies^27–29^, partial removal of HTT protein appears to be well-tolerated^30^. In humans, the tolerability of long-term partial lowering of normal HTT has been demonstrated in SMA children treated with branaplam. Beyond HD, reducing gene expression by triggering pseudoexon formation underscores the potential for disruptive splice modulation as an attractive approach for reversibly downregulating genes associated with disease.

## Methods

### Human RNA-seq data

Libraries were sequenced in paired-end mode, 2 × 76 base pairs (bp; first study) and 2 × 51 bp (washout study) on a llumina NovaSeq 6000 following the Illumina TruSeq Stranded mRNA protocol. The number of reads per sample was between 24 and 32 million for the first study, and 32 and 69 million reads per sample for the washout study. Reads were mapped to the hg38 (GRCh38.p12) genome and to the transcripts from Ensembl (version 94) by using an in-house gene and exon quantification pipeline (EQP with STAR)^13,31^. On average, 98% (first study) and 97% (washout study) of the total reads (depending on the library) were mapped to the genome or the transcripts, and 91% (first study) and 83% (washout study) of the aligned reads mapped to expressed sequences.

### Mouse RNA-seq data

Each library was sequenced in paired-end mode, 2 × 76 bp, using the Illumina HiSeq 2500 platform with Illumina TruSeq RNA Sample Preparation protocol v2. The number of reads per sample was between 52 and 66 million. Reads were mapped to the mm10 genome and to the transcripts from Ensembl (version 87) by using an in-house gene and EQP^31^. On average, 96% of the total reads (depending on the library) were mapped to the genome or the transcripts, and 83% of the aligned reads mapped to expressed sequences.

### Differential gene expression analysis

Differential gene expression analysis was conducted with a script in R (version 3.6.3). The genome and the transcript alignments were used to derive gene counts, which represent the total number of reads aligned to each gene and were then transformed into counts per million (CPM; normalization by the total number of mapped reads per sample) and fragments per kilobase of exon per million of fragments mapped (FPKM; further normalization by effective gene length). After assessment by principal component analysis and multidimensional scaling, all samples were retained for further analysis. Only genes with counts above 0.5 reads per kilobase of transcript per million mapped (RPKM) in at least two samples were included. Differential expression analysis between branaplam vs. dimethyl sulfoxide (DMSO) was performed on the RPKM using a limma/voom workflow with R^32^. Results are reported in terms of log_2_ fold changes and negative log_10_ adjusted *P* values (Benjamini–Hochberg false discovery rate).

### Pseudoexon detection and differential analysis

Fastq files for all six samples are aligned against the human genome hg38 (GRCh38.p12) using the STAR aligner (version 2.7.1a^13^) as STAR predicts introns from gapped alignments. The read counts were combined for the predicted introns over all six samples. Only introns with more than 12 reads in total are retained (the threshold was lowered to 8 reads for the washout experiment in order to account for the shorter read length of 51 bases which lowers the sensitivity for splice junctions). All pairs of intron ends and intron starts that are on the same strand and at least 12 bp apart but no more than 512 bp apart were collected. This was conducted with a script in R (version 3.6.0). The genomic intervals defined by these pairs were intersected with all Ensembl (version 94) exons using bedtools (version 2.27.1). Only intervals that do not intersect with any Ensembl exon were kept as candidate pseudoexons. The sequences of these candidates, plus 5 bp flanks on both sides, were extracted from the genome using bedtools. The candidates were quantified by their read coverage in all six samples using bedtools. The coverage values were then analyzed with a limma/voom based workflow and tested for differential expression between DMSO and branaplam (R version 3.6.0 and Bioconductor 3.10). The 5’-ends (3 + 5) of differentially expressed candidates upregulated by branaplam (adjusted *P* value < 0.01 and log_2_ > 1), and those intersecting with a GENCODE gene (94 candidates in first dataset/138 candidates in the second dataset -- annotation in terms of transcription start and end and gene symbol were downloaded from UCSC table browser in June 2020: GencodeBasicV33) were processed with Bioconductor package seqLogo to visualize the composition. The overlap between the two datasets is 83. Coverage plots for visualization of individual genes were generated with Bioconductor package SGSeq. Disjoint exon models, i.e., a set of nonoverlapping exon bins from all the transcripts of a gene, were generated for each gene using function disjointExons of the R/Bioconductor package GenomicFeatures. For each sample, the number of sequenced fragments overlapping with each exon bin was tabulated and normalized for library size using the DESeq2 method.

### Sequence alignment and exon conservation

Using an R script (version 3.6.1), genomic sequences from the *HTT* gene were retrieved from Ensembl (version 99, Jan 2020). Using the biomaRt library (version 2.40.5, pmid 19617889 and 16082012) sequences were retrieved with the getSequence function [biomaRt::getSequence(id = < ensembl gene id > , type = “ensembl_gene_id”, seqType = “gene_exon_intron”, mart = < species mart > ], with the mart and ensembl gene id listed below for each species. Sequences were retrieved for five species: human (hsapiens_gene_ensembl, ENSG00000197386), mouse (musculus_gene_ensembl, ENSMUSG00000029104), rat (rnorvegicus_gene_ensembl, ENSRNOG00000011073), dog (cfamiliaris_gene_ensembl, ENSCAFG00000014682), and chimpanzee (ptroglodytes_gene_ensembl, ENSPTRG00000015854). A rough alignment of the full gene sequences with ClustalOmega was used to identify sequences proximal to the novel exon within and across species. To create Supplementary Fig. 3, the sequences around 1000 bp of the novel exon were aligned using DECIPHER (version 2.14.0) with the following parameters: iterations = 5, refinements = 5 and gapExtension = c(−15, −12). The alignments were cleaned using the AdjustAlignment function. A subset of the alignment proximal to the splice site was extracted with the subseq function and visualized with the BrowseSeqs function (DECIPHER).

### Snaptron

The novel cassette exon is located at chr4:3213622-3213736 (hg38 coordinates) and the canonical intron that normally splices out the novel cassette exon is located at chr4:3212710-3213957 (hg38) as depicted in Fig. 2c. The Snaptron^17^ server was installed locally, and the full region queried using the parameters snaptron?regions=chr4:3212710-3213957.

The resulting data were processed with R (version 3.6.1) using functions and packages from the Tidyverse^33^ (reader, version 1.3.1; dplyr, version 0.8.3; tidyr, version 1.0.0). The intron upstream of the novel cassette exon was isolated with dplyr::filter(start ==  3212710 & end ==  3213621). The intron downstream of the novel cassette exon was isolated with dplyr::filter(start ==  3213737 & end ==  3213957). The results were pivoted and samples limited to those in common between the upstream and downstream introns to isolate samples that ead to the inclusion of the novel cassette exon. Similarly, the canonical junction was isolated with dplyr::filter(start ==  3212710 & end ==  3213957).

### Cloning of LMI070-regulatable expression vector with an HTT minigene switch

The sequence spanning from exon 49 to exon 50 of the homo sapiens HTT gene (NG_009378.1) was synthesized by Integrated DNA Technologies, Inc. (IDT). The complete sequence of the minigene is provided in Supplementary Table 5. The following modifications were made in the minigene compared with the original HTT genomic sequence: A Kozak consensus sequence and an ATG codon were added upstream of exon 49; two stop codons in LMI070-dependent pseudoexons were mutated to maintain an open reading frame (ORF); a single nucleotide in exon 50 was mutated to create a stop codon; a single nucleotide in exon 50 was removed to maintain OFR with the downstream transgene; three ATG codons in exons 49, 50, and an intron were mutated. The HTT minigene was cloned in a mammalian expression plasmid by *Age*I and *Apa*I sites between the JeT promoter and FurinCS/T2A coding sequence (RNRR GSGEGRGSLLTCGDVEENPGP) that was followed by green fluorescent protein (GFP).

Next, the GFP CDS was replaced by NanoLuc luciferase CDS synthesized by IDT. For altering the LMI070-dependent splice donor, AGA located on the 3’-end of the LMI070-dependent pseudoexon was mutated to AGC, ACA, or CGA by replacing the *Stu*I-*Pst*I fragment with a corresponding fragment containing the mutation (synthesized by IDT).

### In vitro evaluation of branaplam

Cultured human neuroblastoma cells (SH-SY5Y) were treated with branaplam at doses ranging from 5 to 125 nM for 24 h (for transcript evaluation) or 48 h (for protein evaluation). RNA was quantified by a Thermo Fisher Scientific Nanodrop 2000. cDNAs were synthesized from 140 to 400 ng RNA using a Maxima First-strand cDNA synthesis kit (Thermo Fisher Scientific). A mix of oligo dT and random hexamers (Thermo Fisher Scientific) was used in 20 µL reactions at 25 °C for 10 min, 50 °C for 15 min, then 85 °C for 5 min. Quantitative polymerase chain reaction (PCR) was performed using 20 µL of TaqMan™ Fast Advanced master mix (Thermo Fisher Scientific) with 4 µL of cDNA reaction and primers specific for each gene. The PCR steps were as follows: 95 °C for 20 s then 40 cycles of 95 °C for 1 s, and then 55 °C for 20 s. The sequence of primers were: for wild-type (WT) human HTT, forward, 5’-GTCATTTGCACCTTCCTCCT-3’ (SEQ ID No.: 1), reverse, 5’- TGGATCAAATGCCAGGACAG-3’ (SEQ ID No.: 2), probe, 56-FAM/TTG TGA AAT /ZEN/TCG TGG TGG CAA CCC /3IABkFQ/ (SEQ ID No.: 8), for the HTT novel exon, forward, 5’-TCCTGAGAAAGAGAAGGACATTG-3’ (SEQ ID No.: 3), reverse, 5’- CTGTGGGCTCCTGTAGAAATC-3’ (SEQ ID No.: 4), probe, 56-FAM/AAT TCG TGG /ZEN/TGG CAA CCC TTG AGA /3IABkFQ/ (SEQ ID No.: 7).

Relative quantification of gene expression was performed using a 2 − ΔΔCT method. Fold changes in the mRNA expression level were calculated following normalization to human glucuronidase beta (Gusb) as an endogenous reference (Fig. 2a, b).

### Protein analysis in HD patient fibroblasts

Human HD patient fibroblasts GM04723 (19-year-old, female, 72 CAG repeats), and ND31551 (19-year-old, male, 39 CAG repeats) were obtained from the Coriell Institute for Medical Research Cell repository and maintained in DMEM media containing 10% fetal bovine serum (FBS), 1% MEM nonessential amino acids, 1% sodium pyruvate, 1% l-glutamine, and 1% penicillin/streptomycin at 37 °C with 5% CO2. For protein analysis, cells were lysed in RIPA buffer with a protease and phosphatase inhibitor (Thermo Fisher Scientific). The supernatant was obtained by centrifugation for 20 min at 13,000 rpm at 4 °C. Total protein concentration was quantified using a BCA protein Assay (Thermo Fisher Scientific). Samples were resolved in 3–8% tris-acetate or 4–12% bis-tris polyacrylamide gel under reducing condition. Proteins were transferred onto a PVDF membrane and western blot analysis was performed using anti-huntingtin (1HU-4C8, Millipore #MAB2166), anti-polyQ (5TF1-1C2, Millipore #MAB1574), anti-huntingtin (2B7, Coriell), anti-actin (Sigma-Aldrich, #A5316), and anti-vinculin (Bio-Rad Laboratories Inc., #MCA465) antibody. Protein bands were quantified by Image J.

### Transfection of the 293T cell line with LMI070-regulatable HTT minigene switch expression vectors

The AAVpro 293T cell line (Takara Bio USA Inc.) was seeded at 20,000 cells/well (293T) in Dulbecco’s Modified Eagle Medium (Gibco, Thermo Fisher Scientific) supplemented with 10% fetal bovine serum (FBS) in 96-well Corning CellBIND surface plates (Thermo Fisher Scientific). Plates were returned to the incubator for 24 h prior to transfection. The HTT minigene Nluc reporter constructs (pJet-HTT50a-Nluc) with the LMI070-dependent splice-donor site AGA (DL258) and constructs with the 3’-splice-donor site altered to AGC (DL273), ACA (DL274), or CGA (DL275) were transfected into 293T cells in six replicate wells at a final concentration of 4 µg/mL using a Lipofectamine 2000 reagent (Thermo Fisher Scientific). At 4 h after the addition of DNA, Lipofectamine 2000 complexes to the cells, and the transfection mixture was replaced with cell growth medium followed by a 20-h incubation. At 24 h, cells were treated with either 0.1% DMSO (Thermo Fisher Scientific) or 25 nM, 50 nM, or 125 nM branaplam and incubated for an additional 24 h to induce LMI070-dependent splicing of the HTT minigene and Nluc luciferase expression. The Nano**-**Glo Luciferase Assay System (Promega) reagent was used to measure the Nluc luminescence signal in the Corning Costar solid white 96-well microplates (VWR) using the SpectraMax Paradigm microplate reader (Molecular Devices LLC). The assay data were analyzed using the GraphPad PRISM (version 8.1.2), and graphed as means with standard deviations.

### Animals

Animal protocols were approved by The Children’s Hospital of Philadelphia Institutional Animal Care and use Committee. BacHD mice were obtained from an in-house bred BacHD colony derived from mice obtained from Jackson Laboratories (Stock No: 008197, FVB/N-Tg(HTT*97Q)IXwy/J, Bar Harbor, ME, USA). Mice were housed in a temperature-controlled environment on a 12 h light/dark cycle. Food and water were provided ad libitum. Branaplam or vehicle solution was administrated by oral gavage every other day for a total of three or four doses a week for the period of time indicated on each experiment. The amount of branaplam or vehicle administrated by oral gavage to each mouse was based on the weight recorded on the day of dosing.

### Blood collection, and tissue sampling of mice

At different timepoints after the last treatment, blood, and tissue samples were obtained for pharmacokinetic (PK) and pharmacodynamic (PD) analyses.

Blood was obtained via submandibular vein bleeds and collected for RNA extraction (PD analysis) using RNAprotect Animal Blood Tubes (Qiagen), and plasma (PK analysis) using K2EDTA coated tubes (Thermo Fisher Scientific). Cells were removed from the plasma by centrifugation for 10 min at 2000 × *g* at 4 °C, and plasma samples were stored at −80 °C. Following blood collection, mice were given a lethal dose of ketamine/xylazine (10 mg ketamine and 1 mg xylazine in 100 mL sterile water) and perfused with 18 mL of 0.9% cold saline mixed with 2 mL of RNAlater (Ambion) solution for tissue collection. Liver, skeletal muscle, and different brain region samples were flash-frozen in liquid nitrogen and stored at −80 °C.

### Motor function evaluation using narrow beam test

Female mice were tested on the narrow beam after LMI070 treatment. The narrow beam was clear with progressively decreasing width from 30 to 10 mm for a total of one meter to reach an enclosed safety platform. The beam was elevated (40–47 cm) with the mice climbing upward. Day 1 was training, where mice crossed the beam five times with a 30 min inter-trial time. Day 2 was testing with the protocol repeated and the behavior filmed and the number of slips measured by a blind observer (Dunnet et al.^23^). The normality of the distributions was assessed using the Shapiro–Wilk test. Data were analyzed using a Kruskal–Wallis test with Dunn’s multiple comparisons test to compare groups. Outliers outside two standard deviations of their group were removed.

### An open-label study of LMI070 (branaplam) in Type 1 spinal muscular atrophy (SMA)

The effect of branaplam on the expression levels of *HTT* mRNA was assessed in infants with Type I SMA who were enrolled in an open-label multipart first-in-human proof-of-concept study of oral branaplam. The trial (clinicalTrials.gov NCT02268552) is still ongoing and is being conducted in accordance with the principles of the Declaration of Helsinki. The trial protocol was approved by the institutional review board or ethics committee at each investigational site (Committee for Medical Ethics, UZ Gent, Belgium; Ethische Commissie onderzoek UZ Leuven, Belgium; Ethics Committee for Clinical Trials, Sofia, Bulgaria; De Videnskabestiske Kimiteer for Region Hovedstaden, Hilleroed, Denmark; Universitatsklinikum, Essen, Germany; Comitato Etico dell’IRCCS Ospedale Pediatrico Bambino Gesù, Rome, Italy; Comitato Etico IRCCS Regione Lombardia -Sezione Fondazione IRCCS Istituto Neurologico Carlo Besta, Milan, Italy; Komisja Bioetyczna przy Instytucie “Pomnik-Centrum Zdrowia Dziecka” w Warszawie, Warsaw, Poland; Veltishev Scientific Research Clinical Pediatric Institute, Moscow, Russia; Regional Research EC of Volgograd State Medical University, Volgograd, Russia; Children City Clinical Hospital N 9, Ekaterinburg, Russia; Moscow City Independent LEC, Moscow, Russia; Federal Medical Research Centre n.a. V.A. Almazov, St. Petersburg, Russia). The additional exploratory gene expression analyses on Huntingtin (*HTT*) mRNA levels using RNA sample remnants extracted from blood of infants enrolled in the ongoing clinical study LMI070X2201 was reviewed and approved by the Swiss Association of Research Ethics Committees (Ethikkommission Nordwest- und Zentralschweiz EKNZ; BASEC-ID: 2018-02215; Project title: TRI0198 - Extended gene expression analysis in clinical study LMI070X2201). Written informed consent was provided by parents or legal guardians of all participants and covered the additional gene expression analyses on Huntingtin (*HTT*) mRNA levels.

All participants had exactly two copies of the *SMN2* gene, were diagnosed clinically with Type 1 SMA and had symptom onset before 6 months of age, and were up to 182 (part 1) or 180 (part 2) days old at screening. In all, 13 participants were recruited in part 1 and another 25 participants were recruited in part 2. All trial participants received once-weekly doses of branaplam.

From part 1 (p1), samples for *HTT* mRNA analysis were available up to 1541 study days. From part 2 (p2), samples were obtained up to 358 study days. In detail, study TRI0198 comprised the following number of subjects with samples collected at the given study days: baseline (p1: *n* = 7, p2: *n* = 22), day 85 (p1: *n* = 1, p2: *n* = 21), day 176 (p1: *n* = 3, p2: *n* = 21), day 267 (p1: *n* = 4, p2: *n* = 18), day 358 (p1: *n* = 4, p2: *n* = 12), day 449 (p1: *n* = 5), day 540 (p1: *n* = 5), day 631 (p1: *n* = 5), day 722 (p1: *n* = 5), day 813 (p1: *n* = 4), day 904 (p1: *n* = 5), day 995 (p1: *n* = 5), day 1086 (p1: *n* = 7), day 1177 (p1: *n* = 5), day 1268 (p1: *n* = 7), day 1359 (p1: *n* = 4), day 1450 (p1: *n* = 5), day 1541 (p1: *n* = 4).

### RNA extraction and quantitative PCR

Total RNA was extracted using a PAXgene Blood RNA Kit (Qiagen). Total RNA was reverse transcribed to cDNA using random hexamers and the iScript^TM^ Advanced cDNA Synthesis Kit (Bio-Rad Laboratories Inc.). cDNA synthesis was performed according to the manufacturer’s instructions using 100 ng of total RNA as input into a 20 μL cDNA reaction to generate an initial cDNA with a concentration of 5 ng/μL (total RNA equivalents). Finally, the cDNA was subsequently diluted 1/1 with nuclease-free water to generate a final cDNA with a concentration of 2.5 ng/μL (total RNA equivalents). All preparations were carried out on ice. cDNA synthesis was performed on a C1000 Thermal cycler, Reaction Module 96 W Fast (Bio-Rad Laboratories Inc.) using the following conditions: 25 °C for 5 min, 46 °C for 20 min, 95 °C for 1 min and hold at 4 °C. cDNA samples were stored at −20 °C.

Levels of HTT mRNA and pseudoexon-included HTT mRNA were then quantified by PCR using the Bio-Rad Laboratories QX200 droplet digital PCR system. Standard reaction and cycling conditions (95 °C for 10 min; 40 cycles of 94 °C for 30 s and 60 °C for 60 s; and 98 °C for 10 min; hold at 4 °C) and a cDNA input (total RNA equivalent) of 20 ng were applied.

For HTT mRNA levels, the following predesigned quantitative PCR assays purchased from Integrated DNA Technologies, Inc. was used: Assay Hs.PT.58.14833829 with forward primer 5′- GAGACTCATCCAGTACCATCAG-3′, reverse primer 5′-GATGTCAGCTATCTGTCGAGAC-3′, and probe 5′-56-FAM/CGCTTCCAC/ZEN/TTGTCTTCATTCTCCTTGT/3IABkFQ-3′.

A customized quantitative PCR assay was applied to quantify the inclusion of a novel exon into HTT mRNA, with forward primer 5′-TCCTGAGAAAGAGAAGGACATTG-3′, reverse primer 5′-CTGTGGGCTCCTGTAGAAATC-3′, and probe 5′-56-FAM/AATTCGTGG/ZEN/TGGCAACCCTTGAGA/3IABkFQ-3′.

All gene expression values were normalized to glucuronidase beta (GUSB) mRNA levels. A predesigned quantitative PCR assay purchased from Integrated DNA Technologies, Inc. was used to assess GUSB mRNA levels. Assay Hs.PT.39a.22214857, with forward primer 5′-TCACTGAAGAGTACCAGAAAAGTC-3′, reverse primer 5′-TTTTATTCCCCAGCACTCTCG-3′, and probe 5′-HEX/ACGCAGAAA/ZEN/ATACGTGGTTGGAGAGC/3IABkFQ-3′.

### Reporting summary

Further information on research design is available in the Nature Research Reporting Summary linked to this article.

## Supplementary information

Supplementary Information

Peer Review File

Reporting Summary

## Data Availability

The data supporting the findings of this study are available from the corresponding authors upon reasonable request. The RNA-seq data generated or analyzed during the current study are available in the NCBI Sequence Read Archive under the following accessions: PRJNA788994, PRJNA638877, and PRJNA639047. For human RNA-seq data, reads were mapped to the hg38 (GRCh38.p12) genome and to the transcripts from Ensembl (version 94). For mouse RNA-seq data, reads were mapped to the mm10 genome and to the transcripts from Ensembl (version 87). Pseudoexon annotation in terms of transcription start and end and gene symbol were downloaded from UCSC table browser in June 2020: GencodeBasicV33.  Source data are provided with this paper.

## Source data

Source Data
