# Supplementary material for: An orally available, brain penetrant, small molecule lowers huntingtin levels by enhancing pseudoexon inclusion
Source: Nat Commun. 2022 Mar 3;13:1150. doi: 10.1038/s41467-022-28653-6 (PMC8894458; doi:10.1038/s41467-022-28653-6)
Supplement: Supplementary file 1 — Supplementary Information [file 41467_2022_28653_MOESM1_ESM.pdf]

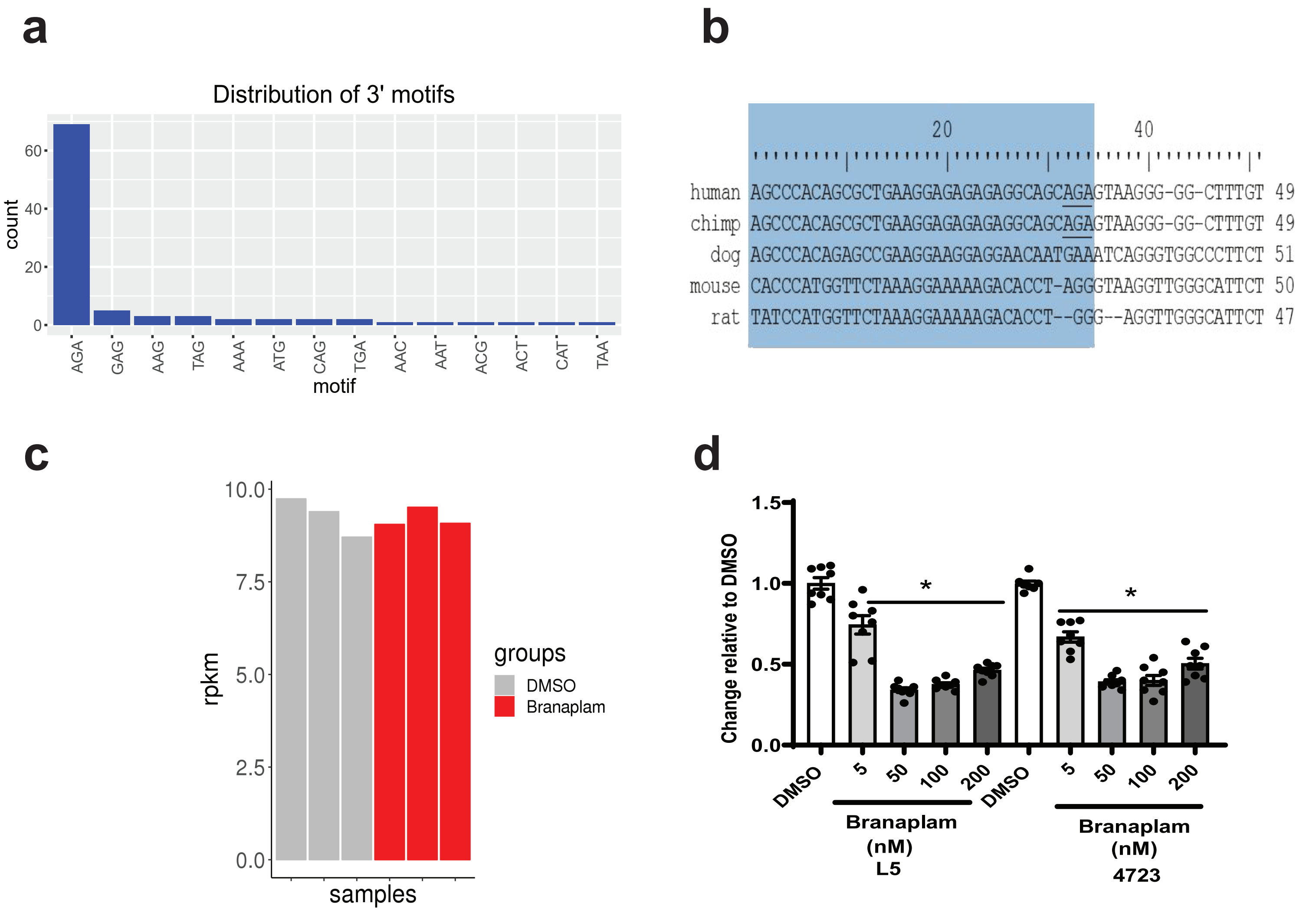

Supplementary Data Figure 1. Sequence alignment and conservation of the 3' exonic motif and modulation of mouse and human *HTT* transcripts by branaplam**. a,** Histogram of 3' ends (XXX|[last 3 bases exonic]) for the 94 differential pseudoexons in Figure 1B that map to GENCODE genes **b,** Alignment and conservation of the sequence of the novel HTT exon across human, chimpanzee, dog, mouse, and rat species. Blue shading indicates putative exonic sequences. **c,** Mouse Htt transcript expression values from RNA-seq experiments in NSC34 cells following DMSO (grey) and branaplam (red) treatments. **d,** Quantitative PCR results showing total HTT mRNA expression levels after DMSO or Branaplam treatment (at indicated doses) for 24 hrs. Two independent HD patient fibroblast cell lines (GM04723 (4723) and ND31551 (L5)), and a taqman assay priming for human HTT exon 1-2 was used. Samples are normalized to human GAPDH, and data are the mean ± SEM relative to HD fibroblasts treated with DMSO (4 biological replicates, *P < 0.001, one-way ANOVA followed by a Bonferroni's post hoc).

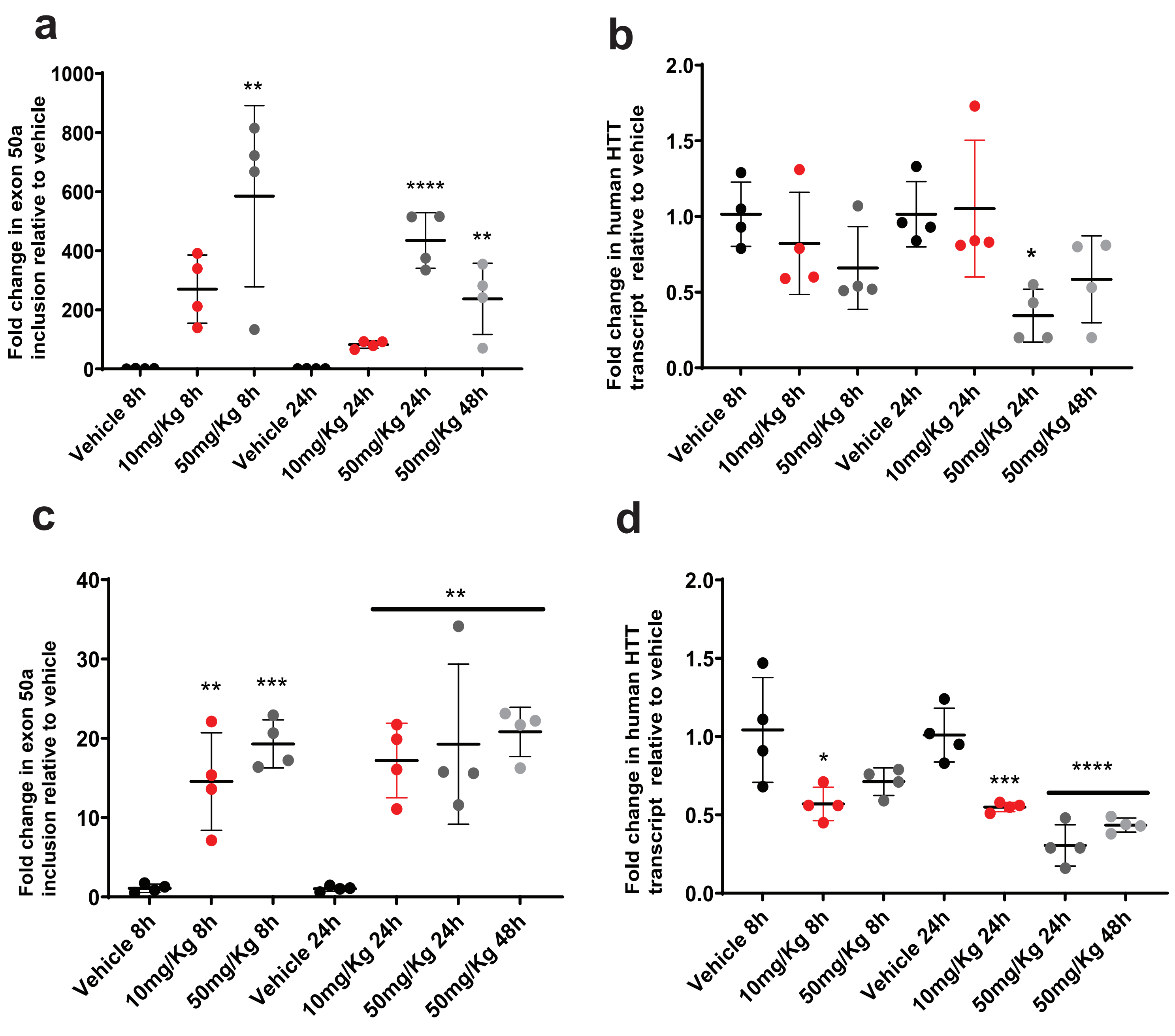

Supplementary Data Figure 2. Modulation of *HTT* transcripts inthe brain and blood of BacHD mice after a single oral dose of branaplam. **a,** pseudoexon 50a included transcript in the cerebrum of BacHD mice (n=4) treated with 10 mg /kg or 50 mg/kg of branaplam and taken down at indicated timepoints. Data are the mean±SEM of 4 mice per group. One-way Anova with Dunnett's multiple comparisons test: **P = 0.0034 or 0.0020, ****P < 0.0001. **b**, human *HTT* transcript levels in the cerebrum of BacHD mice (n=4) treated with 10 mg /kg or 50 mg/kg of branaplam and taken down at indicated timepoints. Data are the mean±SEM of 4 mice per group. One-way Anova with Dunnett's multiple comparisons test: *P = 0.6700. **c,** pseudoexon 50a included transcript levels from blood of BacHD mice (n = 4) treated with a single dose of 10 mg/kg or 50 mg/kg of branaplam analyzed at the indicated timepoints. Data are the mean±SEM of 4 mice per group. One-way Anova with Dunnett's multiple comparisons test: **P = 0.0018, ***P = 0.0002 and **d**, human *HTT* transcript levels from blood of BacHD mice (n=4) treated with 10 mg /kg or 50 mg/kg of branaplam and taken down at indicated timepoints. Data are the mean±SEM of 4 mice per group. One-way Anova with Dunnett's multiple comparisons test: *P = 0.0198, ***P = 0.0002, ****P < 0.0001.

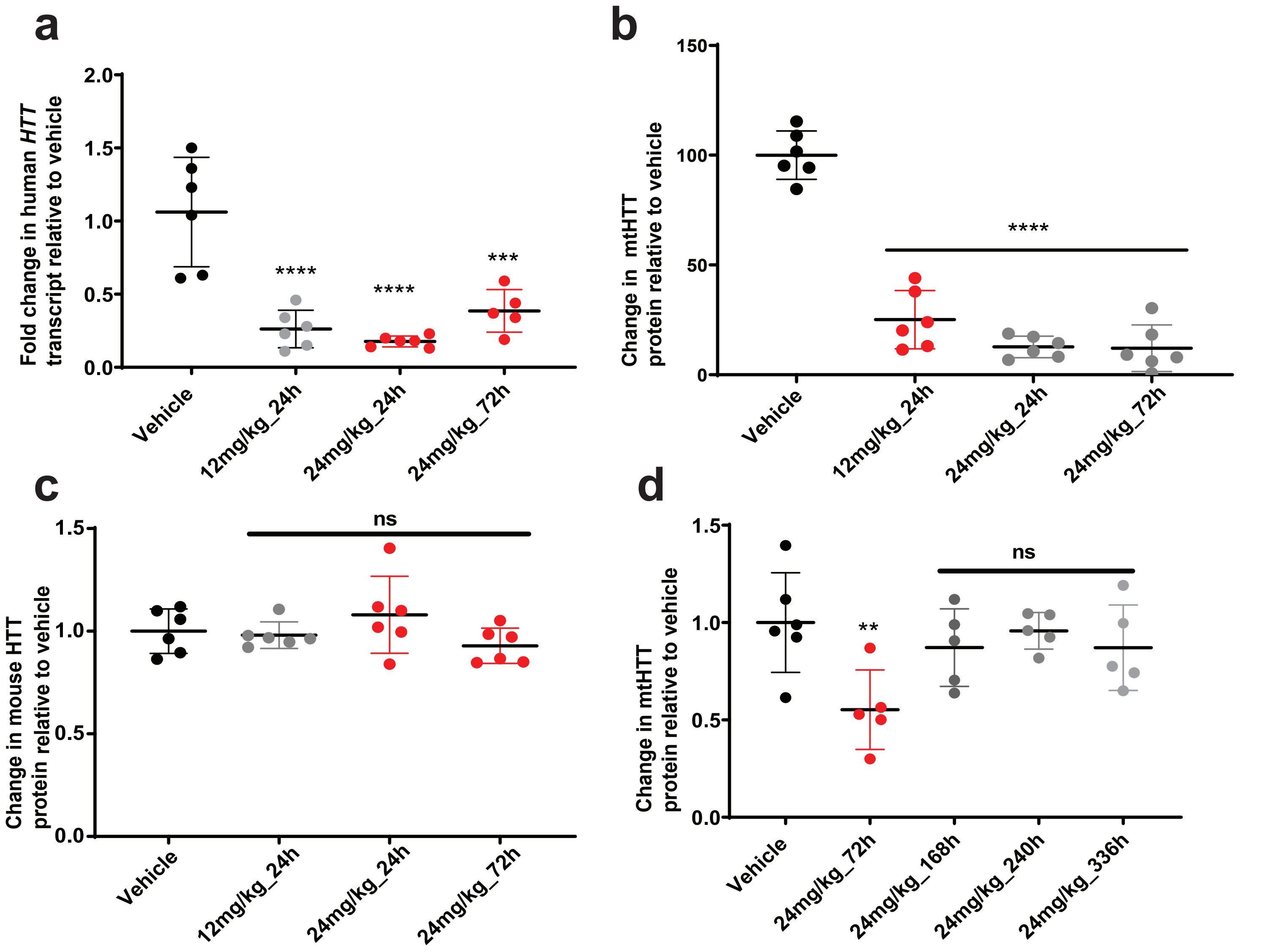

Supplementary Data Figure 3. Peripheral and brain *HTT* transcript and protein changes in BacHD mice treated with branaplam. **a,** Quantitative PCR results showing total *HTT* transcript levels in the blood of BacHD mice treated with vehicle or branaplam (n = 6). Mice received either 12 mg/kg or 24 mg/kg of branaplam for 3 weeks and blood was analyzed at indicated timepoints. Data are the mean±SEM of 6 mice per group. One-way Anova with Dunnett's multiple comparisons test: **** $P < 0.0001$. **b,** mtHTT protein levels in the liver of BacHD mice (n = 6) treated with 12 mg /kg or 24 mg/kg of branaplam and taken down at indicated timepoints. Data are the mean±SEM of 6 mice per group. One-way Anova with Dunnett's multiple comparisons test: **** $P < 0.0001$. **c,** Mouse Htt protein levels in striatum from BacHD mice treated with vehicle or branaplam (n=6) at 12mg/kg or 24 mg/kg and taken down at indicated doses. Data are the mean±SEM of 6 mice per group. None of the branaplam treated conditions were significantly different compared to vehicle. **d,** Time-course of mtHTT protein levels in the cortex of BacHD mice (n = 6) treated with 12 mg/kg or 24 mg/kg of branaplam and taken down at indicated timepoints. Data are the mean±SEM of 6 mice per group. One-way Anova with Dunnett's multiple comparisons test: ** $P = 0.0060$.

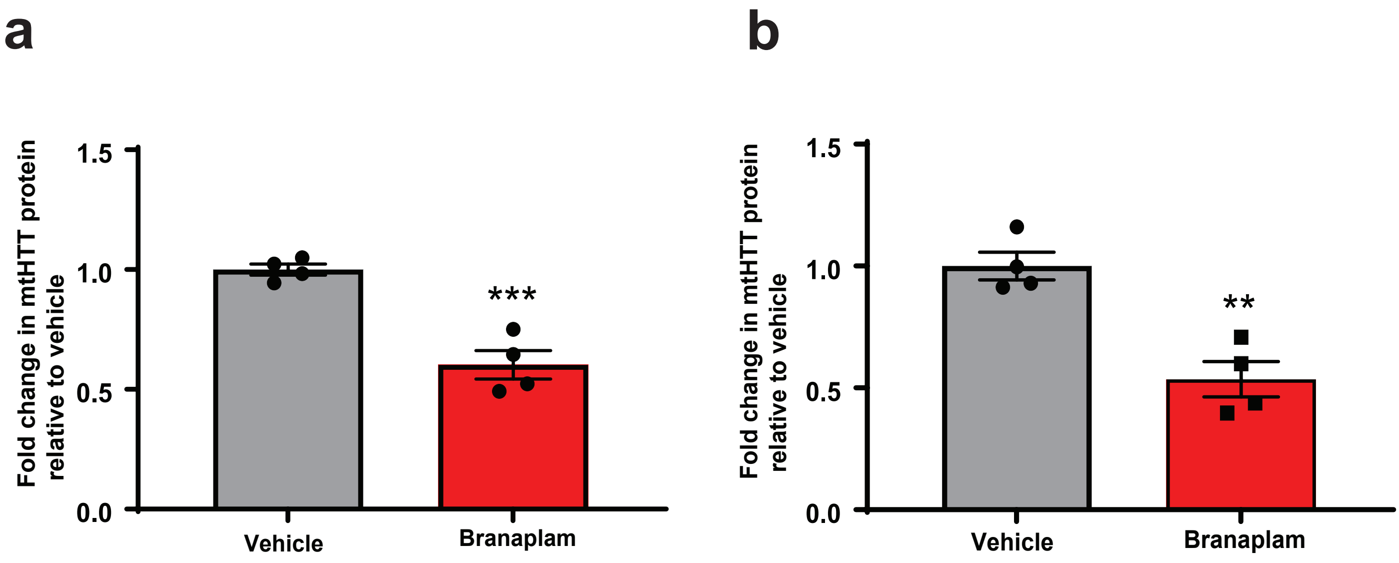

Supplementary Data Figure 4. mHTT protein lowering in BacHD mice brain following oral dosing with branaplam **a,** mHTT protein levels in the striatum of BacHD mice (n = 4) treated with 16 doses of 24mg/kg of branaplam. Data is the mean±SEM of 4 mice per group. P values in this study were calculated by unpaired t test (Two-tailed Unpaired t-Test: ***P = 0.0008 **b,** mHTT protein levels in the cortex of BacHD mice (n = 4) treated with 16 doses of 24mg/kg of branaplam. Data is the mean±SEM of 4 mice per group. P values in this study were calculated by unpaired t test (Two-tailed). Unpaired t-Test: **P = 0.0023.

Supplementary Data Table 1

SH-SY5Y cells) treated with 100 nM branaplam for 24 hours. A total of 45 genes exhibited an absolute fold change > 2 with a multiple testing correction (MTC)-adjusted *P* value < 0.01. Statistical analysis was performed with limma/voom. P values are two-sided and multiplicity adjusted.

Ensembl Gene ID	Description	hgnc Symbol	$\log_{FC}$	adj. P value
ENSG00000180957	phosphatidylinositol transfer protein beta	PITPNB	-2.90671	3.41E-07
ENSG00000088930	5'-3' exoribonuclease 2	XRN2	-2.69807	1.04E-06
ENSG00000139734	diaphanous related formin 3	DIAPH3	-1.71324	3.01E-06
ENSG00000179889	pyridoxal dependent decarboxylase domain containing 1	PDXDC1	-2.85615	3.01E-06
ENSG00000228716	dihydrofolate reductase	DHFR	-1.63086	3.01E-06
ENSG00000156052	G protein subunit alpha q	GNAQ	-2.01613	4.10E-06
ENSG00000137710	radixin	RDX	-1.23828	6.04E-06
ENSG00000163507	cellular inhibitor of PP2A	CIP2A	-1.49306	6.04E-06
ENSG00000119537	3-ketodihydrosphingosine reductase	KDSR	-1.69007	1.84E-05
ENSG00000153914	splicing regulatory glutamic acid and lysine rich protein 1	SREK1	-1.31531	1.84E-05
ENSG00000164329	terminal nucleotidyltransferase 2	TENT2	-1.32786	1.84E-05
ENSG00000197386	huntingtin	HTT	-1.07408	1.90E-05
ENSG00000113643	arginyl-tRNA synthetase 1	RARS1	-1.09349	2.23E-05
ENSG00000112146	F-box protein 9	FBXO9	-1.39965	4.00E-05
ENSG00000103064	solute carrier family 7 member 6	SLC7A6	-1.10575	4.30E-05
ENSG00000156298	tetraspanin 7	TSPAN7	-2.04839	4.30E-05
ENSG00000134775	formin homology 2 domain containing 3	FHOD3	-1.17727	4.31E-05
ENSG00000162687	potassium sodium-activated channel subfamily T member 2	KCNT2	-1.40537	4.31E-05
ENSG00000180198	regulator of chromosome condensation 1	RCC1	1.24293	4.31E-05
ENSG00000250349	novel proline rich Gla (G-carboxyglutamic acid) 1 (PRRG1) and tetraspanin 7 (TSPAN7) protein		-2.04137	4.31E-05
ENSG00000110881	acid sensing ion channel subunit 1	ASIC1	-2.13038	4.69E-05
ENSG00000101464	phosphatidylinositol glycan anchor biosynthesis class U	PIGU	-1.05053	4.99E-05
ENSG00000144320	lunapark, ER junction formation factor	LNPK	-1.09676	5.93E-05
ENSG00000064933	PMS1 homolog 1, mismatch repair system component	PMS1	-1.30932	6.90E-05
ENSG00000137501	synaptotagmin like 2	SYTL2	3.871721	6.90E-05
ENSG00000054983	galactosylceramidase	GALC	-1.5173	0.000108
ENSG00000100395	L3MBTL histone methyl-lysine binding protein 2	L3MBTL2	-1.37989	0.000108
ENSG00000109911	elongator acetyltransferase complex subunit 4	ELP4	-1.32842	0.00014
ENSG00000120471	tumor protein p53 regulated apoptosis inducing protein 1	TP53AIP1	4.210064	0.000149
ENSG00000160392	chromosome 19 open reading frame 47	C19orf47	-1.10856	0.000149
ENSG00000273820	ubiquitin specific peptidase 27 X-linked	USP27X	-1.72153	0.000187
ENSG00000122477	leucine rich repeat containing 39	LRRC39	2.429588	0.000232
ENSG00000007171	nitric oxide synthase 2	NOS2	1.400148	0.000278
ENSG00000184014	DENN domain containing 5A	DENND5A	-1.00993	0.000288
ENSG00000143196	dermatopontin	DPT	-1.77668	0.000637
ENSG00000103145	host cell factor C1 regulator 1	HCFC1R1	-1.1029	0.000804
ENSG00000152475	zinc finger protein 837	ZNF837	1.862342	0.000804
ENSG00000179284	DAN domain BMP antagonist family member 5	DAND5	-1.18702	0.000857

ENSG00000142188	transmembrane protein 50B	TMEM50B	-1.05588	0.001238
ENSG00000198162	mannosidase alpha class 1A member 2	MAN1A2	-1.09818	0.001844
ENSG00000127585	F-box and leucine rich repeat protein 16	FBXL16	-1.15242	0.003286
ENSG00000165475	crystallin lambda 1	CRYL1	-1.75215	0.003989
ENSG00000167774	novel transcript		-1.01957	0.005332
ENSG00000105427	cornifelin	CNFN	-2.23858	0.006539
ENSG00000156345	cyclin dependent kinase 20	CDK20	-1.20958	0.009429

Supplementary Data Table 2

SH-SY5Y cells) treated with 100 nM branaplam for 24 hours. A total of 94 pseudoexons map to GENCODE genes and show an absolute fold change > 2 and a multiple testing correction (MTC)-adjusted *P* value < 0.01. Statistical analysis was performed with limma/voom. P values are two-sided and multiplicity adjusted.

Exon ID	log $_{FC}$	Adj. *P* value	Symbol	Chr	Start	End
chr16_70527375_70527429	8.02112	3.34E-11	SF3B3	16	70527375	70527429
chr5_77774184_77774217	7.83225	8.86E-10	TBCA	5	77774184	77774217
chr1_98738659_98738803	7.6699	3.34E-11	SNX7	1	98738659	98738803
chr12_4537379_4537514	7.32822	2.06E-10	C12orf4	12	4537379	4537514
chr12_895160_895196	6.91717	1.24E-10	WNK1	12	895160	895196
chr18_55535558_55535637	6.81571	2.69E-10	TCF4	18	55535558	55535637
chr8_47273336_47273450	6.70626	2.06E-10	SPIDR	8	47273336	47273450
chr2_8820966_8821102	6.66208	1.63E-09	KIDINS220	2	8820966	8821102
chr5_119457290_119457368	6.4788	3.35E-09	HSD17B4	5	119457290	119457368
chr6_97201361_97201466	6.34876	8.29E-10	MMS22L	6	97201361	97201466
chr17_3647737_3647847	6.34502	1.49E-10	CTNS	17	3647737	3647847
chr16_24758438_24758599	6.33985	3.35E-09	TNRC6A	16	24758438	24758599
chr10_21728631_21728675	6.14493	2.57E-10	MLLT10	10	21728631	21728675
chr2_27037261_27037300	6.14159	8.54E-10	TMEM214	2	27037261	27037300
chr7_101948854_101948970	6.07441	2.90E-10	CUX1	7	101948854	101948970
chr12_40869291_40869394	5.93415	6.16E-09	CNTN1	12	40869291	40869394
chr12_40869295_40869394	5.93415	6.16E-09	CNTN1	12	40869295	40869394
chr2_2055809_2056066	5.89886	7.32E-10	MYT1L	2	2055809	2056066
chr2_201470906_201471111	5.84198	5.78E-09	STRADB	2	201470906	201471111
chr5_51545529_51545767	5.83233	6.38E-09	AC022433.1	5	51545529	51545767
chr13_72725641_72725778	5.82948	3.03E-09	MZT1	13	72725641	72725778
chr4_5741333_5741441	5.79592	8.86E-10	EVC	4	5741333	5741441
chr19_36400284_36400403	5.77568	3.17E-09	ZFP82	19	36400284	36400403
chr14_32150458_32150566	5.54797	3.17E-09	ARHGAP5	14	32150458	32150566
chr7_133794224_133794324	5.54064	4.37E-09	EXOC4	7	133794224	133794324
chr18_36742376_36742468	5.51839	5.53E-08	FHOD3	18	36742376	36742468
chr3_108192475_108192526	5.49749	3.17E-09	IFT57	3	108192475	108192526
chr22_21578780_21578838	5.42634	2.57E-09	UBE2L3	22	21578780	21578838
chr16_70544168_70544249	5.30709	3.14E-08	SF3B3	16	70544168	70544249
chr7_82067208_82067256	5.29629	1.78E-08	CACNA2D1	7	82067208	82067256
chr10_77812712_77812773	5.26504	1.76E-08	DLG5	10	77812712	77812773
chr4_102853037_102853083	5.25868	4.37E-09	UBE2D3	4	102853037	102853083
chr1_84105240_84105388	5.25453	7.35E-08	PRKACB	1	84105240	84105388
chr1_84105245_84105388	5.25453	7.35E-08	PRKACB	1	84105245	84105388
chr5_122898141_122898236	5.24644	9.22E-08	SNX24	5	122898141	122898236
chr3_49397004_49397122	5.21737	1.50E-08	RHOA	3	49397004	49397122
chr12_39441961_39442087	5.2108	2.79E-09	KIF21A	12	39441961	39442087
chr8_22643371_22643742	5.09941	2.60E-08	BIN3	8	22643371	22643742
chr21_37422581_37422652	5.09026	2.51E-08	DYRK1A	21	37422581	37422652
chr6_110984306_110984363	5.07173	1.76E-08	RPF2	6	110984306	110984363
chr21_31268410_31268695	5.0307	1.57E-07	TIAM1	21	31268410	31268695
chr16_70544168_70544207	4.96945	8.15E-08	SF3B3	16	70544168	70544207
chr9_77920661_77920702	4.95553	1.76E-08	GNAQ	9	77920661	77920702

chr8_116861153_116861297	4.95166	3.87E-07	RAD21	8	116861153	116861297
chr7_137001862_137002057	4.95107	3.35E-09	CHRM2	7	137001862	137002057
chr2_31887034_31887087	4.94023	2.60E-07	MEMO1	2	31887034	31887087
chr11_76548927_76549017	4.86385	2.52E-08	EMSY	11	76548927	76549017
chr19_8814260_8814371	4.80908	6.58E-08	ZNF558	19	8814260	8814371
chr5_80632231_80632302	4.79421	6.53E-09	DHFR	5	80632231	80632302
chr12_42095150_42095214	4.71684	2.04E-07	GXYLT1	12	42095150	42095214
chr6_100636927_100636984	4.69586	4.82E-08	ASCC3	6	100636927	100636984
chr9_123454002_123454051	4.67422	4.82E-08	DENND1A	9	123454002	123454051
chr12_20602224_20602321	4.63816	1.04E-07	PDE3A	12	20602224	20602321
chr17_38312586_38312661	4.638	3.42E-07	MRPL45	17	38312586	38312661
chr7_43905371_43905451	4.63788	4.55E-07	URGCP-MRPS24,URGCP	7	43905371	43905451
chr2_75702708_75702807	4.5743	3.23E-06	GCFC2	2	75702708	75702807
chr4_3213621_3213736	4.44684	4.82E-08	HTT	4	3213621	3213736
chr1_35020260_35020279	4.44672	6.58E-08	ZMYM6, AC114490.2	1	35020260	35020279
chr6_75621926_75621971	4.3645	2.05E-07	SENP6	6	75621926	75621971
chr10_68680871_68680967	4.31289	1.81E-06	TET1	10	68680871	68680967
chr7_64541729_64541917	4.30358	3.40E-07	ZNF680	7	64541729	64541917
chrX_101156521_101156555	4.27255	6.14E-07	CENPI	X	101156521	101156555
chr2_6973931_6974023	4.25244	8.87E-07	RNF144A	2	6973931	6974023
chr6_169661702_169661750	4.17289	3.15E-06	AL031315.1, WDR27	6	169661702	169661750
chr19_39836893_39836973	4.0321	2.45E-07	FBL	19	39836893	39836973
chr6_170544320_170544366	4.00533	6.20E-06	PSMB1	6	170544320	170544366
chr9_133109193_133109278	3.9624	1.99E-07	AL162417.1,RALGDS	9	133109193	133109278
chr1_37494139_37494163	3.93041	4.73E-06	MEAF6	1	37494139	37494163
chr10_68907910_68907970	3.72181	6.18E-06	DDX50	10	68907910	68907970
chr9_125964037_125964198	3.66576	4.91E-07	PBX3	9	125964037	125964198
chr1_211304989_211305151	3.6514	6.86E-07	RCOR3	1	211304989	211305151
chr3_108565897_108565931	3.54176	9.04E-05	CIP2A	3	108565897	108565931
chr2_110638984_110639069	3.51955	3.65E-05	BUB1	2	110638984	110639069
chr4_112638617_112638673	3.39139	3.87E-07	LARP7	4	112638617	112638673
chr19_14173295_14173399	3.38153	8.79E-05	ADGRL1	19	14173295	14173399
chr17_65015912_65016040	3.30508	6.84E-06	GNA13	17	65015912	65016040
chr1_225600207_225600362	3.28521	8.86E-10	ENAH	1	225600207	225600362
chr20_21345833_21345887	3.27557	1.13E-06	XRN2	20	21345833	21345887
chr9_123452825_123452908	3.22454	7.46E-06	DENND1A	9	123452825	123452908
chr14_66987935_66988007	3.10933	0.00025058	GPHN	14	66987935	66988007
chr3_108565897_108566078	2.99823	0.000487262	CIP2A	3	108565897	108566078
chr2_36884519_36884606	2.74115	3.20E-05	STRN	2	36884519	36884606
chr19_45691162_45691293	2.71347	0.00052116	SNRPD2	19	45691162	45691293
chr2_8810660_8810778	2.64457	3.79E-05	KIDINS220	2	8810660	8810778
chr5_79641454_79641517	2.58341	6.39E-05	TENT2	5	79641454	79641517
chr8_79631016_79631127	2.52256	1.62E-05	STMN2	8	79631016	79631127
chr3_108566041_108566078	2.47737	0.002060581	CIP2A	3	108566041	108566078
chr10_75230410_75230450	2.37965	0.0001598	VDAC2	10	75230410	75230450
chr11_75602633_75602718	2.34905	0.00021672	MAP6	11	75602633	75602718
chr3_125470565_125470661	2.34275	0.000155183	SNX4	3	125470565	125470661
chr5_71038428_71038481	2.06474	0.000663706	GTF2H2	5	71038428	71038481

chr4_41749199_41749342	1.93646	7.47E-06	AC105389.3	4	41749199	41749342
chr2_68315707_68315843	1.92446	6.59E-05	CNRIP1	2	68315707	68315843
chr8_65721146_65721297	1.90499	0.001182493	PDE7A	8	65721146	65721297

Supplementary Data Table 3

SH-SY5Y cells) treated with 100 nM branaplam for 24 hours in a washout experiment. A total of 69 genes exhibited an absolute fold change > 2 with a multiple testing correction (MTC)-adjusted *P* value < 0.01. Statistical analysis was performed with limma/voom. P values are two-sided and multiplicity adjusted.

Ensembl Gene ID	Description	hgnc Symbol	log_{FC}	adj. P value
ENSG00000088930	5'-3' exoribonuclease 2	XRN2	-2.99093	6.09E-09
ENSG00000179889	pyridoxal dependent decarboxylase domain containing 1	PDXDC1	-3.24261	6.09E-09
ENSG00000180957	phosphatidylinositol transfer protein beta	PITPNB	-3.35755	2.31E-08
ENSG00000100395	L3MBTL histone methyl-lysine binding protein 2	L3MBTL2	-1.78873	3.82E-07
ENSG00000119537	3-ketodihydrosphingosine reductase	KDSR	-2.05296	1.60E-06
ENSG00000054983	galactosylceramidase	GALC	-1.82013	3.36E-06
ENSG00000112146	F-box protein 9	FBXO9	-1.98915	5.65E-06
ENSG00000137501	synaptotagmin like 2	SYTL2	4.006548	7.86E-06
ENSG00000184014	DENN domain containing 5A	DENND5A	-1.43631	8.17E-06
ENSG00000071794	helicase like transcription factor	HLTF	-1.22284	8.61E-06
ENSG00000137710	radixin	RDX	-1.49585	1.86E-05
ENSG00000160392	chromosome 19 open reading frame 47	C19orf47	-1.38572	1.86E-05
ENSG00000163507	cellular inhibitor of PP2A	CIP2A	-1.90559	1.86E-05
ENSG00000103064	solute carrier family 7 member 6	SLC7A6	-1.29953	2.18E-05
ENSG00000103145	host cell factor C1 regulator 1	HCFC1R1	-1.38967	2.18E-05
ENSG00000153914	splicing regulatory glutamic acid and lysine rich protein 1	SREK1	-1.70588	2.18E-05
ENSG00000198162	mannosidase alpha class 1A member 2	MAN1A2	-1.64448	2.20E-05
ENSG00000008277	ADAM metallopeptidase domain 22	ADAM22	-1.60432	2.79E-05
ENSG00000105427	cornifelin	CNFN	-2.80441	2.79E-05
ENSG00000134775	formin homology 2 domain containing 3	FHOD3	-1.35916	2.95E-05
ENSG00000064933	PMS1 homolog 1, mismatch repair system component	PMS1	-1.64507	3.23E-05
ENSG00000115970	THADA armadillo repeat containing	THADA	-1.09815	3.52E-05
ENSG00000156052	G protein subunit alpha q	GNAQ	-2.28653	3.90E-05
ENSG00000162959	mediator of cell motility 1	MEMO1	-1.19117	3.90E-05
ENSG00000166938	DIS3 like exosome 3'-5' exoribonuclease	DIS3L	-1.18676	3.90E-05
ENSG00000109911	elongator acetyltransferase complex subunit 4	ELP4	-1.42475	4.29E-05
ENSG00000197386	huntingtin	HTT	-1.32846	4.29E-05
ENSG00000228716	dihydrofolate reductase	DHFR	-1.97679	4.29E-05
ENSG00000273820	ubiquitin specific peptidase 27 X-linked	USP27X	-1.67403	4.56E-05
ENSG00000164329	terminal nucleotidyltransferase 2	TENT2	-1.28309	5.18E-05
ENSG00000090372	striatin 4	STRN4	-1.1077	5.60E-05
ENSG00000139734	diaphanous related formin 3	DIAPH3	-2.04821	7.68E-05
ENSG00000168803	adenosine deaminase like	ADAL	-1.1489	8.25E-05
ENSG00000180198	regulator of chromosome condensation 1	RCC1	1.652608	8.25E-05
ENSG00000151468	coiled-coil domain containing 3	CCDC3	-1.82671	8.32E-05
ENSG00000110881	acid sensing ion channel subunit 1	ASIC1	-2.93288	8.39E-05
ENSG00000143702	centrosomal protein 170	CEP170	-1.41616	9.13E-05
ENSG00000151208	discs large MAGUK scaffold protein 5	DLG5	-1.10008	9.69E-05
ENSG00000113643	arginyl-tRNA synthetase 1	RARS1	-1.23982	0.000127

ENSG00000127585	F-box and leucine rich repeat protein 16	FBXL16	-1.49953	0.000132
ENSG00000018510	alkylglycerone phosphate synthase	AGPS	-1.39242	0.000132
ENSG00000156298	tetraspanin 7	TSPAN7	-2.23282	0.000265
ENSG00000070785	eukaryotic translation initiation factor 2B subunit gamma	EIF2B3	-1.21753	0.000288
ENSG00000170962	platelet derived growth factor D	PDGFD	-1.50681	0.000288
ENSG00000007171	nitric oxide synthase 2	NOS2	1.197153	0.000367
ENSG00000143196	dermatopontin	DPT	-2.63858	0.000382
ENSG00000119522	DENN domain containing 1A	DENND1A	-1.10337	0.00042
ENSG00000153317	ArfGAP with SH3 domain, ankyrin repeat and PH domain 1	ASAP1	-1.18256	0.00042
ENSG00000105202	fibrillarin	FBL	-1.02063	0.000553
ENSG00000101773	RB binding protein 8, endonuclease	RBBP8	-1.00639	0.000699
ENSG00000115556	phospholipase C delta 4	PLCD4	-1.13521	0.001271
ENSG00000152475	zinc finger protein 837	ZNF837	1.416362	0.001271
ENSG00000100234	TIMP metallopeptidase inhibitor 3	TIMP3	-1.13682	0.001538
ENSG00000158286	ring finger protein 207	RNF207	-1.38223	0.001974
ENSG00000156345	cyclin dependent kinase 20	CDK20	-1.91468	0.002142
ENSG00000162687	potassium sodium-activated channel subfamily T member 2	KCNT2	-1.42879	0.002166
ENSG00000204334	glutamate rich 2	ERICH2	3.482546	0.002984
ENSG00000170627	gametocyte specific factor 1	GTSF1	-1.03755	0.003058
ENSG00000132570	pterin-4 alpha-carbinolamine dehydratase 2	PCBD2	-1.02002	0.003874
ENSG00000250349	novel proline rich Gla (G-carboxyglutamic acid) 1 (PRRG1) and tetraspanin 7 (TSPAN7) protein		-1.99722	0.003917
ENSG00000145194	endothelin converting enzyme 2	ECE2	-2.93703	0.003961
ENSG00000122477	leucine rich repeat containing 39	LRRC39	1.742848	0.00433
ENSG00000140470	ADAM metallopeptidase with thrombospondin type 1 motif 17	ADAMTS17	-1.23943	0.005732
ENSG00000083642	PDS5 cohesin associated factor B	PDS5B	-1.00518	0.006438
ENSG00000157734	sorting nexin 22	SNX22	-1.20121	0.007196
ENSG00000149084	hydroxysteroid 17-beta dehydrogenase 12	HSD17B12	-1.00209	0.007454
ENSG00000176658	myosin ID	MYO1D	-1.07417	0.007454
ENSG00000136205	tensin 3	TNS3	-2.0102	0.007887
ENSG00000165475	crystallin lambda 1	CRYL1	-1.74565	0.007935

Supplementary Data Table 4

SH-SY5Y cells) treated with 100 nM branaplam for 24 hours in a washout experiment. A total of 138 pseudoexons map to GENCODE genes and show an absolute fold change > 2 and a multiple testing correction (MTC)-adjusted *P* value < 0.01. The two pseudoexon-gene pairs not reversed after 48 hours post-washout are marked with an asterix. Statistical analysis was performed with limma/voom. P values are two-sided and multiplicity adjusted.

exon ID	log FC	adj.P.Val	symbol	chr	start	end
chr1_98738659_98738803	9.46006	4.18E-09	SNX7	1	98738659	98738803
chr16_70527375_70527429	8.80107	2.96E-09	SF3B3	16	70527375	70527429
chr12_4537379_4537514	8.1412	4.59E-09	C12orf4	12	4537379	4537514
chr5_51545529_51545767	8.02606	2.63E-09	AC022433.1	5	51545529	51545767
chr5_77774184_77774217*	8.02298	7.51E-10	TBCA	5	77774184	77774217
chr13_72725641_72725778	7.82707	2.63E-09	MZT1	13	72725641	72725778
chr2_201470906_201471111	7.74574	3.06E-08	STRADB	2	2.01E+08	2.01E+08
chr5_119457290_119457368	7.71537	2.96E-09	HSD17B4	5	1.19E+08	1.19E+08
chr18_55535558_55535637	7.40754	2.63E-09	TCF4	18	55535558	55535637
chr8_22643371_22643742	7.37381	2.63E-09	BIN3	8	22643371	22643742
chr6_97201361_97201466	7.02411	4.51E-09	MMS22L	6	97201361	97201466
chr16_70544168_70544249	7.00002	1.56E-07	SF3B3	16	70544168	70544249
chr2_27037261_27037300	6.98892	4.51E-08	TMEM214	2	27037261	27037300
chr12_40869295_40869394	6.94602	2.78E-08	CNTN1	12	40869295	40869394
chr12_895160_895196	6.82617	4.51E-09	WNK1	12	895160	895196
chr1_84105240_84105388	6.76157	3.60E-07	PRKACB	1	84105240	84105388
chr1_84105245_84105388	6.76157	3.60E-07	PRKACB	1	84105245	84105388
chr14_32150458_32150566	6.72464	9.93E-09	ARHGAP5	14	32150458	32150566
chr8_47273336_47273450	6.70715	1.78E-08	SPIDR	8	47273336	47273450
chr2_8820966_8821102	6.70656	2.52E-08	KIDINS220	2	8820966	8821102
chr16_70544168_70544207	6.67739	2.14E-07	SF3B3	16	70544168	70544207
chr10_21728631_21728675	6.66321	1.78E-08	MLLT10	10	21728631	21728675
chr22_21578780_21578838	6.59205	1.39E-07	UBE2L3	22	21578780	21578838
chr10_77812712_77812773	6.55859	1.00E-06	DLG5	10	77812712	77812773
chr21_37422581_37422652	6.48272	6.61E-09	DYRK1A	21	37422581	37422652
chr8_22643651_22643742	6.37355	1.12E-08	BIN3	8	22643651	22643742
chr12_39441961_39442087	6.34541	1.45E-07	KIF21A	12	39441961	39442087
chr9_77920661_77920702	6.32624	4.51E-08	GNAQ	9	77920661	77920702
chr6_110984306_110984363	6.31835	1.94E-07	RPF2	6	1.11E+08	1.11E+08
chr17_3647737_3647847	6.26696	1.78E-08	CTNS	17	3647737	3647847
chr1_44318140_44318224	6.18443	5.40E-07	ERI3	1	44318140	44318224
chr3_108192475_108192526	6.17288	3.56E-07	IFT57	3	1.08E+08	1.08E+08
chr7_43905371_43905451	6.1575	4.51E-08	URGCP,URGCP-MRPS24	7	43905371	43905451
chr3_49397004_49397122	6.13413	1.47E-07	RHOA	3	49397004	49397122
chr3_49397020_49397122	6.12546	1.47E-07	RHOA	3	49397020	49397122
chr9_123454002_123454051	6.06606	5.55E-08	DENND1A	9	1.23E+08	1.23E+08
chr19_14173295_14173399	6.0367	1.69E-07	ADGRL1	19	14173295	14173399
chr16_24758438_24758599	6.02789	3.61E-08	TNRC6A	16	24758438	24758599

chr4_102853037_102853083	6.01483	1.39E-07	UBE2D3	4	1.03E+08	1.03E+08
chr21_31268410_31268695	5.97196	1.35E-07	TIAM1	21	31268410	31268695
chr19_36400284_36400403	5.93222	3.13E-08	ZFP82	19	36400284	36400403
chr2_171441858_171442036	5.85479	5.05E-08	DCAF17	2	1.71E+08	1.71E+08
chr1_184446151_184446247	5.84901	3.57E-08	C1orf21	1	1.84E+08	1.84E+08
chr2_31887034_31887087	5.8209	3.42E-08	MEMO1	2	31887034	31887087
chr7_133794224_133794324	5.64436	4.49E-08	EXOC4	7	1.34E+08	1.34E+08
chr2_75702690_75702807	5.64124	1.73E-07	GCFC2	2	75702690	75702807
chr2_64112562_64112672	5.63083	7.59E-07	PELI1	2	64112562	64112672
chr2_75702708_75702807	5.62967	1.73E-07	GCFC2	2	75702708	75702807
chr5_111750351_111750425	5.55037	8.12E-07	NREP	5	1.12E+08	1.12E+08
chr2_6973931_6974023	5.52931	8.14E-08	RNF144A	2	6973931	6974023
chr17_38312586_38312661	5.51276	3.60E-07	MRPL45	17	38312586	38312661
chr4_5741333_5741441	5.49251	1.73E-07	EVC	4	5741333	5741441
chr7_101948854_101948970	5.43449	8.15E-08	CUX1	7	1.02E+08	1.02E+08
chr17_2031899_2031943	5.2438	5.66E-07	DPH1	17	2031899	2031943
chr22_35267367_35267514	5.19672	1.68E-05	HMGXB4	22	35267367	35267514
chr1_35020260_35020279	5.18851	1.68E-05	ZMYM6,AC114490.2	1	35020260	35020279
chr11_76548927_76549017	5.15707	1.41E-06	EMSY	11	76548927	76549017
chr8_116861153_116861297	5.11232	2.97E-06	RAD21	8	1.17E+08	1.17E+08
chr7_64541729_64541917	5.10935	3.50E-07	ZNF680	7	64541729	64541917
chr9_133109193_133109278	4.99446	3.14E-05	AL162417.1,RALGDS	9	1.33E+08	1.33E+08
chr1_66585671_66585848	4.98692	2.13E-05	SGIP1	1	66585671	66585848
chr17_50720828_50720880	4.86219	2.36E-06	LUC7L3	17	50720828	50720880
chr11_13419276_13419343	4.81325	2.51E-06	BTBD10	11	13419276	13419343
chr5_80632231_80632302	4.78762	1.49E-07	DHFR	5	80632231	80632302
chr4_22447329_22447356	4.77113	3.75E-05	ADGRA3	4	22447329	22447356
chr9_125964037_125964198	4.69762	0.000108	PBX3	9	1.26E+08	1.26E+08
chrX_101156521_101156555	4.66155	3.39E-06	CENPI	X	1.01E+08	1.01E+08
chr10_101848399_101848474	4.65801	1.62E-05	ARMH3	10	1.02E+08	1.02E+08
chr6_75621926_75621971	4.58977	6.28E-07	SENP6	6	75621926	75621971
chr4_3213621_3213736 *	4.54014	1.41E-06	HTT	4	3213621	3213736
chr4_56325387_56325471	4.4685	2.04E-05	CRACD	4	56325387	56325471
chr14_66987935_66988007	4.39703	7.80E-05	GPHN	14	66987935	66988007
chr10_70166275_70166393	4.30071	2.82E-05	SAR1A	10	70166275	70166393
chr3_138206674_138206717	4.26948	1.71E-05	ARMC8	3	1.38E+08	1.38E+08
chr12_42095150_42095214	4.26006	1.03E-05	GXYLT1	12	42095150	42095214
chr5_78647681_78647738	4.25315	2.44E-05	LHFPL2	5	78647681	78647738
chr2_189818737_189818829	4.17121	0.000626	PMS1	2	1.90E+08	1.90E+08
chr10_45459739_45459877	4.15122	3.73E-05	MARCHF8	10	45459739	45459877
chr1_154262172_154262202	4.11869	8.99E-06	UBAP2L	1	1.54E+08	1.54E+08
chr15_55195664_55195822	4.10566	2.93E-06	RSL24D1	15	55195664	55195822
chr5_78647681_78647811	4.09647	4.74E-05	LHFPL2	5	78647681	78647811
chr1_37494139_37494163	4.07451	5.61E-05	MEAF6	1	37494139	37494163
chr1_161870971_161871061	4.0647	2.34E-05	ATF6	1	1.62E+08	1.62E+08
chr3_108565897_108565931	4.00725	0.000206	CIP2A	3	1.09E+08	1.09E+08
chr10_75230410_75230450	3.98156	3.61E-08	VDAC2	10	75230410	75230450

chr5_72283504_72283658	3.9621	0.00044	MRPS27	5	72283504	72283658
chr15_55195746_55195822	3.93617	2.00E-05	RSL24D1	15	55195746	55195822
chr9_123452825_123452908	3.90631	1.28E-05	DENND1A	9	1.23E+08	1.23E+08
chr5_175819435_175819490	3.90071	0.000365	CPLX2	5	1.76E+08	1.76E+08
chr1_211304989_211305151	3.88785	7.36E-05	RCOR3	1	2.11E+08	2.11E+08
chr19_8814260_8814371	3.8827	0.000113	ZNF558	19	8814260	8814371
chr7_137001862_137002057	3.84545	5.44E-09	CHRM2	7	1.37E+08	1.37E+08
chr18_62097680_62097764	3.79201	0.000349	PIGN	18	62097680	62097764
chr6_84222977_84223068	3.664	0.000299	CEP162	6	84222977	84223068
chr18_63340935_63341080	3.65373	4.12E-05	KDSR	18	63340935	63341080
chr14_22571878_22571964	3.62852	0.000108	DAD1	14	22571878	22571964
chr8_79631016_79631127	3.60492	1.78E-08	STMN2	8	79631016	79631127
chr11_75601909_75602029	3.58614	0.000206	MAP6	11	75601909	75602029
chr6_169661702_169661750	3.58204	0.000142	WDR27,AL031315.1	6	1.70E+08	1.70E+08
chr20_21345833_21345887	3.56607	5.70E-05	XRN2	20	21345833	21345887
chr6_170544320_170544366	3.48503	0.00102	PSMB1	6	1.71E+08	1.71E+08
chr1_225600207_225600362	3.44889	5.97E-13	ENAH	1	2.26E+08	2.26E+08
chr19_18927243_18927359	3.33144	0.00102	DDX49	19	18927243	18927359
chr19_45691162_45691293	3.32648	1.45E-06	SNRPD2	19	45691162	45691293
chr4_112638617_112638673	3.29674	8.05E-08	LARP7	4	1.13E+08	1.13E+08
chr2_241347515_241347637	3.24557	0.000142	SEPTIN2	2	2.41E+08	2.41E+08
chr6_100636927_100636984	3.23514	0.000976	ASCC3	6	1.01E+08	1.01E+08
chr9_14089321_14089359	3.08527	0.00044	NFIB	9	14089321	14089359
chr17_65015912_65016040	2.99584	3.17E-05	GNA13	17	65015912	65016040
chr19_39836893_39836973	2.94087	0.00012	FBL	19	39836893	39836973
chr4_41749199_41749342	2.88688	1.12E-08	AC105389.3	4	41749199	41749342
chr10_68907910_68907970	2.81238	0.00061	DDX50	10	68907910	68907970
chr2_68313301_68313413	2.65845	0.001681	CNRIP1	2	68313301	68313413
chr5_78647437_78647738	2.49524	1.76E-06	LHFPL2	5	78647437	78647738
chr7_26206607_26206696	2.47537	0.002489	CBX3	7	26206607	26206696
chr5_78647437_78647811	2.4747	1.50E-06	LHFPL2	5	78647437	78647811
chr2_8810660_8810778	2.43983	0.001871	KIDINS220	2	8810660	8810778
chr7_100706497_100706600	2.38446	0.006755	POP7	7	1.01E+08	1.01E+08
chr19_54139275_54139513	2.36807	0.001716	CNOT3	19	54139275	54139513
chr11_57557234_57557485	2.36625	0.001378	UBE2L6	11	57557234	57557485
chr8_65721146_65721297	2.35887	0.000112	PDE7A	8	65721146	65721297
chr19_54128736_54128805	2.33387	0.000172	PRPF31	19	54128736	54128805
chr5_79641454_79641517	2.27739	0.001907	TENT2	5	79641454	79641517
chr12_2860048_2860145	2.26066	0.000668	FOXM1	12	2860048	2860145
chr5_141668470_141668651	2.16367	0.002511	ARAP3	5	1.42E+08	1.42E+08
chr3_125470565_125470661	2.14864	1.94E-05	SNX4	3	1.25E+08	1.25E+08
chr5_78647437_78647574	2.06432	3.69E-05	LHFPL2	5	78647437	78647574
chr3_108565897_108566078	1.96501	0.007946	CIP2A	3	1.09E+08	1.09E+08
chr5_71038428_71038481	1.91463	0.000344	GTF2H2	5	71038428	71038481
chr5_148411079_148411238	1.86666	0.003135	FBXO38	5	1.48E+08	1.48E+08
chr8_79635093_79635253	1.83864	0.001239	STMN2	8	79635093	79635253
chr2_68315707_68315843	1.76243	3.83E-05	CNRIP1	2	68315707	68315843

chr4_99159420_99159622	1.73747	0.001846	AP002026.1	4	99159420	99159622
chr12_105231368_105231644	1.72493	6.28E-07	APPL2	12	1.05E+08	1.05E+08
chr5_16068415_16068837	1.22332	0.000122	MARCHF11	5	16068415	16068837
chr5_16068415_16068867	1.22023	0.00012	MARCHF11	5	16068415	16068867
chr5_16068415_16068908	1.20195	0.00012	MARCHF11	5	16068415	16068908
chrX_118028935_118029098	1.12301	0.001382	KLHL13	X	1.18E+08	1.18E+08

Table 5

**Animal Species**	**Dose**	**Mean plasma concentration**	**Tissue / Plasma ratio of mean drug concentrations of drug for defined brain regions**		
		**(ng/mL)**	**Frontal cortex**	**Putamen**	**Caudate nucleus**
**NHP**[a]	0.6 mg/kg/BiW [a]	11.3	7.81	8.94	7.52
	3 mg/kg/BiW [a]	74.5	7.57	7.64	6.07
	6 mg/kg/BiW [a]	102	9.79	10.6	8.53
**Dog**[b]	0.25 mg/kg/QD [b]	0.500	19.1	na	12.6
	2 mg/kg/QD [b]	6.23	6.84	na	6.28

a: Tissue and plasma samples collected at 24 h after last dosing of 6 weeks of bi-weekly administration

b: Tissue and plasma samples collected at 24 h after last dosing at Day 7 of daily administration
