# Supplementary material for: An orally available, brain penetrant, small molecule lowers huntingtin levels by enhancing pseudoexon inclusion
Source: Nat Commun. 2022 Mar 3;13:1150. doi: 10.1038/s41467-022-28653-6 (PMC8894458; doi:10.1038/s41467-022-28653-6)
Supplement: Supplementary file 3 — Reporting Summary [file 41467_2022_28653_MOESM3_ESM.pdf]

nature research

Corresponding author(s): Rajeev Sivasankaran
Last updated by author(s): Dec 24, 2021

Reporting Summary

Nature Research wishes to improve the reproducibility of the work that we publish. This form provides structure for consistency and transparency in reporting. For further information on Nature Research policies, see our Editorial Policies and the Editorial Policy Checklist.

Statistics

For all statistical analyses, confirm that the following items are present in the figure legend, table legend, main text, or Methods section.

n/a	Confirmed	
☐	☒	The exact sample size (*n*) for each experimental group/condition, given as a discrete number and unit of measurement
☐	☒	A statement on whether measurements were taken from distinct samples or whether the same sample was measured repeatedly
☐	☒	The statistical test(s) used AND whether they are one- or two-sided<br>*Only common tests should be described solely by name; describe more complex techniques in the Methods section.*
☐	☒	A description of all covariates tested
☐	☒	A description of any assumptions or corrections, such as tests of normality and adjustment for multiple comparisons
☐	☒	A full description of the statistical parameters including central tendency (e.g. means) or other basic estimates (e.g. regression coefficient) AND variation (e.g. standard deviation) or associated estimates of uncertainty (e.g. confidence intervals)
☐	☒	For null hypothesis testing, the test statistic (e.g. *F*, *t*, *r*) with confidence intervals, effect sizes, degrees of freedom and *P* value noted<br>*Give P values as exact values whenever suitable.*
☒	☐	For Bayesian analysis, information on the choice of priors and Markov chain Monte Carlo settings
☒	☐	For hierarchical and complex designs, identification of the appropriate level for tests and full reporting of outcomes
☒	☐	Estimates of effect sizes (e.g. Cohen's *d*, Pearson's *r*), indicating how they were calculated

Our web collection on statistics for biologists contains articles on many of the points above.

Software and code

Policy information about availability of computer code

Data collection	The RNA-seq data was collected using the bcl2fastq Conversion Software.
Data analysis	For human RNA-seq data, reads were mapped to the hg38 (GRCh38.p12) genome and to the transcripts from Ensembl (version 94). For mouse RNA-seq data, reads were mapped to the mm10 genome and to the transcripts from Ensembl (version 87). Pseudoexon annotation in terms of transcription start and end and gene symbol were downloaded from UCSC table browser in June 2020: GencodeBasicV33. The code and processed data to reproduce the analyses presented here are included in this published article (see compressed Extended folder) and are written in the R programing language (version 3.6.0, 3.6.1 and 3.6.3 as indicated in the method sections).

For manuscripts utilizing custom algorithms or software that are central to the research but not yet described in published literature, software must be made available to editors and reviewers. We strongly encourage code deposition in a community repository (e.g. GitHub). See the Nature Research guidelines for submitting code & software for further information.

Data

Policy information about availability of data

All manuscripts must include a data availability statement. This statement should provide the following information, where applicable:

- Accession codes, unique identifiers, or web links for publicly available datasets
- A list of figures that have associated raw data
- A description of any restrictions on data availability

The RNA-seq data generated and / or analyzed during the current study are available in the NCBI Sequence Read Archive under the following accessions: PRJNA788994 (https://www.ncbi.nlm.nih.gov/sra/?term=PRJNA788994), PRJNA638877 (https://www.ncbi.nlm.nih.gov/sra/?term=PRJNA638877), and

PRJNA639047 (https://www.ncbi.nlm.nih.gov/sra/?term=PRJNA639047).PRJNA788994 (https://www.ncbi.nlm.nih.gov/sra/?term=PRJNA788994), PRJNA638877 (https://www.ncbi.nlm.nih.gov/sra/?term=PRJNA638877), and PRJNA639047 (https://www.ncbi.nlm.nih.gov/sra/?term=PRJNA63904).

Field-specific reporting

Please select the one below that is the best fit for your research. If you are not sure, read the appropriate sections before making your selection.

☒ Life sciences ☐ Behavioural & social sciences ☐ Ecological, evolutionary & environmental sciences

For a reference copy of the document with all sections, see nature.com/documents/nr-reporting-summary-flat.pdf

Life sciences study design

All studies must disclose on these points even when the disclosure is negative.

Sample size	Sample sizes for each experiment are indicated in the figure legends. No specific statistical methods were used to estimate sample size. Sample sizes for experiments were based on previous experience using similar experimental setups that showed significance.
Data exclusions	Data were not excluded from the analysis
Replication	The number of biological or technical replicates are indicated in the figure legends for each experiment.
Randomization	The experiments were not randomized.
Blinding	Investigators were not blinded to study to account for appropriate handling and data acquisition.

Reporting for specific materials, systems and methods

We require information from authors about some types of materials, experimental systems and methods used in many studies. Here, indicate whether each material, system or method listed is relevant to your study. If you are not sure if a list item applies to your research, read the appropriate section before selecting a response.

Materials & experimental systems

n/a	Involved in the study	
☐	☒	Antibodies
☐	☒	Eukaryotic cell lines
☒	☐	Palaeontology and archaeology
☐	☒	Animals and other organisms
☐	☒	Human research participants
☐	☒	Clinical data
☒	☐	Dual use research of concern

Methods

n/a	Involved in the study	
☒	☐	ChIP-seq
☒	☐	Flow cytometry
☒	☐	MRI-based neuroimaging

Antibodies

Antibodies used	Rabbit anti-huntingtin antibody (Millipore #MAB2166, Clone 1HU4C8), mouse anti-actin (Sigma-Aldrich, #A5316, Clone AC-74) , mouse anti-vinculin (BioRad Laboratories Inc, #MCA465, Clone V284). Secondary antibodies from Cell Sungaling Technoloy- Anti-mouse IgG, HRP-linked Antibody #7076<br>Anti-rabbit IgG, HRP-linked Antibody #7074<br><br>Ms anti-huntingtin antibody<br>Millipore, #MAB2166, clone 1HU-4C8 (1:1000 dilution used)<br>Ms anti-polyQ antibody<br>Millipore, #MAB1574, clone 5TF1-1C2 (1:1000 dilution used)<br>Ms anti-huntingtin antibody<br>Coriell Institute, # CH03023, clone 2B7 (1:2000 dilution used)<br>Ms anti-vinculin antibody<br>Bio-Rad, # MCA465GA, clone V284 (1:1000 dilution used)<br>This vinculin antibody has been widely used for loading control for western blot analysis<br>Ms anti-actin antibody<br>Sigma-Aldrich, #A5316, clone AC-74 (1:10000 dilution used)<br>Ms anti- α-tubulin antibody<br>Sigma-Aldrich, #T6199, clone DM1A (1:10000 dilution used)
Validation	The anti-huntingtin antibody has been used extensively in the field. Couple of example publications:<br>Acetylation Targets Mutant Huntingtin to Autophagosomes for Degradation, Cell (2009)

The cryo-electron microscopy structure of huntingtin, Nature (2018)
The anti-polyQ antibody (1C2) has been used extensively in the field.

Example publications:
Assessing average somatic CAG repeat instability at the protein level, Nature (2019)
N17 Modifies Mutant Huntingtin Nuclear Pathogenesis and Severity of Disease in HD BAC Transgenic Mice, Neuron (2015)

Generation and validation of 2B7 antibody:
Single-step detection of mutant huntingtin in animal and human tissues: a bioassay for Huntington's disease, Anal Biochem (2009)

This antibody has been used for mHTT detection in mHTT lowering clinical studies:
Validation of Ultrasensitive Mutant Huntingtin Detection in Human Cerebrospinal Fluid by Single Molecule Counting Immunoassay, J. Huntingtons Dis (2017)
Quantification of mutant huntingtin protein in cerebrospinal fluid from Huntington's disease patients, J. Clin. Invest (2015)

This vinculin antibody has been widely used as a loading control in western blot analyses
Example publications:
Serine residues 726 and 780 have nonredundant roles regulating STAT5a activity in luminal breast cancer, Nature (2021)
mTOR Attenuation with Rapamycin Reverses Neurovascular Uncoupling and Memory Deficits in Mice Modeling Alzheimer's Disease, J. Neuroscience (2021)

This actin antibody has been widely used for loading control for western blot analysis
Example publications:
NUAK2 is a critical YAP target in liver cancer, Nature (2018)
Smad2 and Smad3 have differential sensitivity in relaying TGFβ signaling and inversely regulate early lineage specification, Nature (2016)

This tubulin antibody has widely been used as a loading control for western blot analyses
Example publications:
GSK-3 and CK2 Kinases Converge on Timeless to Regulate the Master Clock, Cell (2016)
Modulating FKBP5/FKBP51 and autophagy lowers HTT (huntingtin) levels, Autophagy (2021)

Eukaryotic cell lines

Policy information about cell lines

Cell line source(s)

SH-SY5Y cell line from ATCC. Human HD patient fibroblasts GM04723 (19 year old, female, 72 CAG repeats), and ND31551(19 year old, male, 39 CAG repeats) were obtained from the Coriell institute for Medical Research Cell repository

Authentication

Cell lines used have been extensively used in the field and were not independently authenticated

Mycoplasma contamination

All cell lines tested negative for mycoplasma

Commonly misidentified lines
(See ICLAC register)

None of the cells used in the study were listed in ICLAC database of commonly misidentified cell lines.

Animals and other organisms

Policy information about studies involving animals; ARRIVE guidelines recommended for reporting animal research

Laboratory animals

BacHD mice were obtained from an in house bred BacHD colony derived from mice obtained from Jackson Laboratories (Stock No: 008197, FVB/N-Tg(HTT*97Q)IXwy/J, Bar Harbor, ME, USA). Mice were housed in a temperature-controlled environment on a 12 hr light/dark cycle. Food and water were provided ad libitum. Male and female BacHD mice of ages ranging from 2-5 months were used for the described studies.

Wild animals

The study did not involve wild animals.

Field-collected samples

The study did not involve field-collected samples.

Ethics oversight

All animal procedures were approved by the CHOP IACUC.

Note that full information on the approval of the study protocol must also be provided in the manuscript.

Human research participants

Policy information about studies involving human research participants

Population characteristics

The effect of branaplam on the expression levels of HTT mRNA was assessed in infants with Type I SMA who were enrolled in an open-label multi-part first-in-human proof of concept study of oral branaplam. The trial (clinicalTrials.gov NCT02268552) is

	still ongoing and is being conducted in accordance with the principles of the Declaration of Helsinki. All participants had exactly 2 copies of the SMN2 gene, were diagnosed clinically with Type 1 SMA and had symptom onset before 6 months of age and were up to 182 (Part 1) or 180 (Part 2) days old at screening. 13 participants were recruited in Part 1 and another 25 participants were recruited in Part 2.
Recruitment	The data included in the current Gubser-Keller et al manuscript is on the analysis of HTT gene expression in SMA Type I patients enrolled in an ongoing clinical trial (LMI070X2201). The authorization for the evaluation of HTT gene expression from these samples are detailed in the Ethics Oversight section below. There was no separate recruitment of patients for the current evaluation.
Ethics oversight	The trial protocol and all documentation were approved by the institutional review board or ethics committee at each investigational site. Written informed consent was provided by parents or legal guardians of all participants. The additional exploratory gene expression analyses on Huntingtin (HTT) mRNA levels (the data which is being reported in the current manuscript) using RNA sample remnants extracted from blood of infants enrolled in the ongoing clinical study LMI070X2201 was reviewed and approved by the Swiss Association of Research Ethics Committees (Ethikkommission Nordwest- und Zentralschweiz EKNZ; BASEC-ID: 2018-02215; Project title: TRI0198 - Extended gene expression analysis in clinical study LMI070X2201).

Note that full information on the approval of the study protocol must also be provided in the manuscript.

Clinical data

Policy information about clinical studies
All manuscripts should comply with the ICMJE guidelines for publication of clinical research and a completed CONSORT checklist must be included with all submissions.

Clinical trial registration	We are not reporting clinical trial data in this manuscript. The data included in the manuscript specifically are on the exploratory gene expression analysis of HTT transcripts from SMA patients enrolled in an ongoing clinical study (LMI070X2201).
Study protocol	*Note where the full trial protocol can be accessed OR if not available, explain why.*
Data collection	*Describe the settings and locales of data collection, noting the time periods of recruitment and data collection.*
Outcomes	*Describe how you pre-defined primary and secondary outcome measures and how you assessed these measures.*
