# Supplementary figures and images for: An orally available, brain penetrant, small molecule lowers huntingtin levels by enhancing pseudoexon inclusion
Source: Nat Commun. 2022 Mar 3;13:1150. doi: 10.1038/s41467-022-28653-6 (PMC8894458; doi:10.1038/s41467-022-28653-6)

Fig. 3c

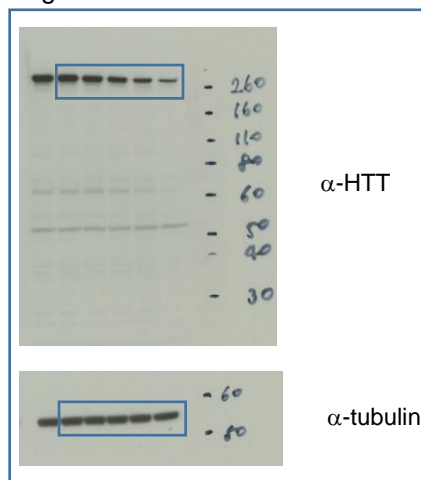

Fig. 3g

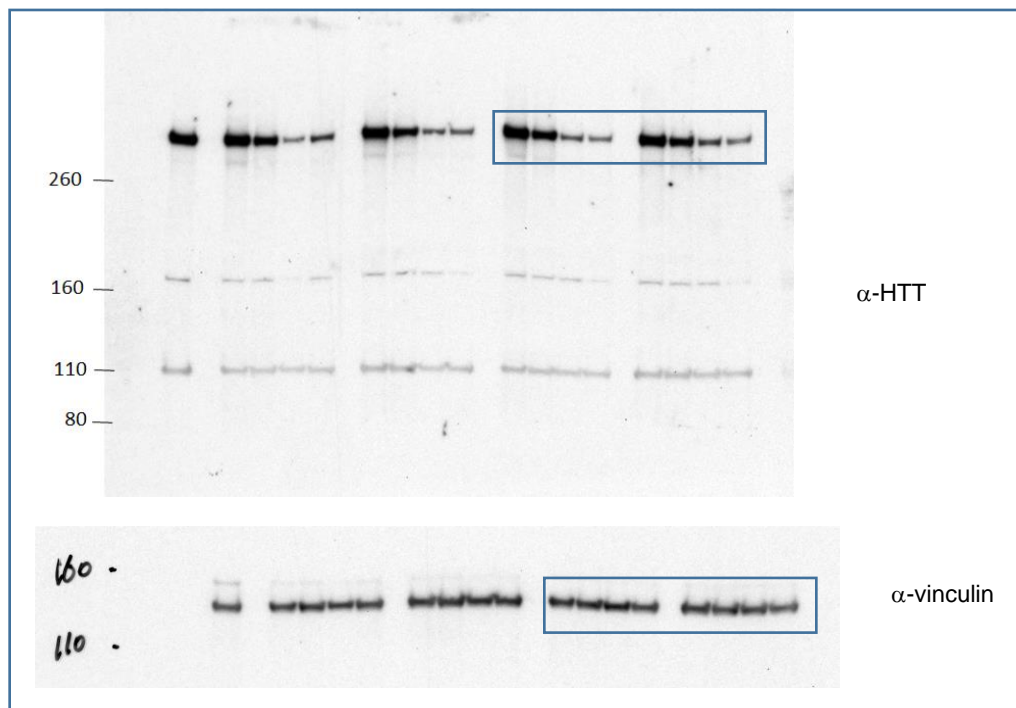

Fig. 4e

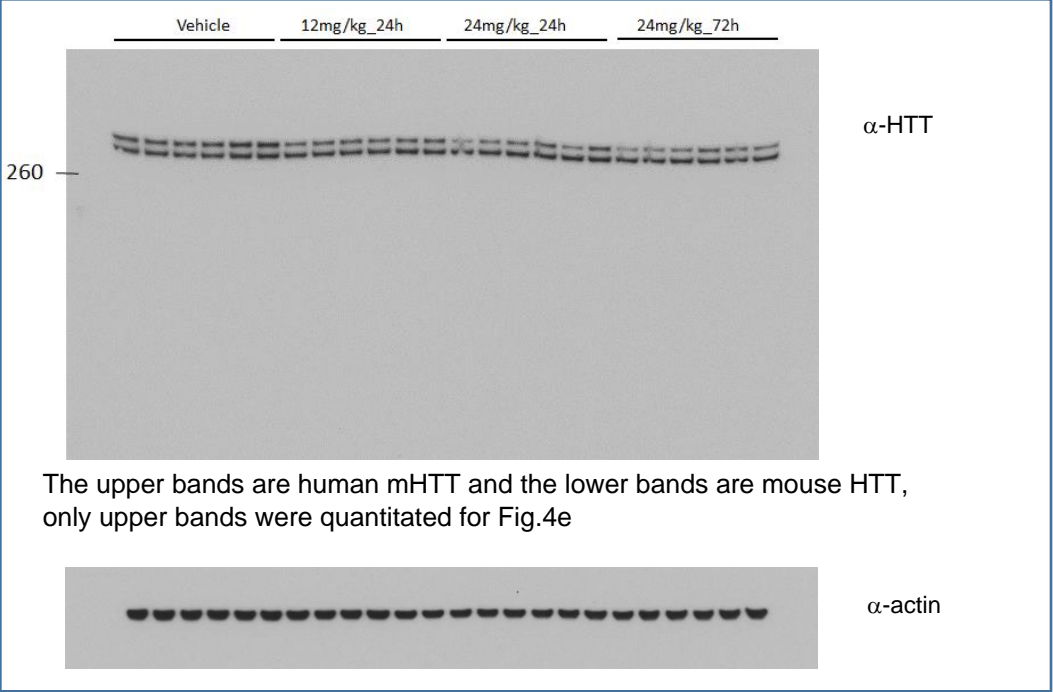

Fig. 4f

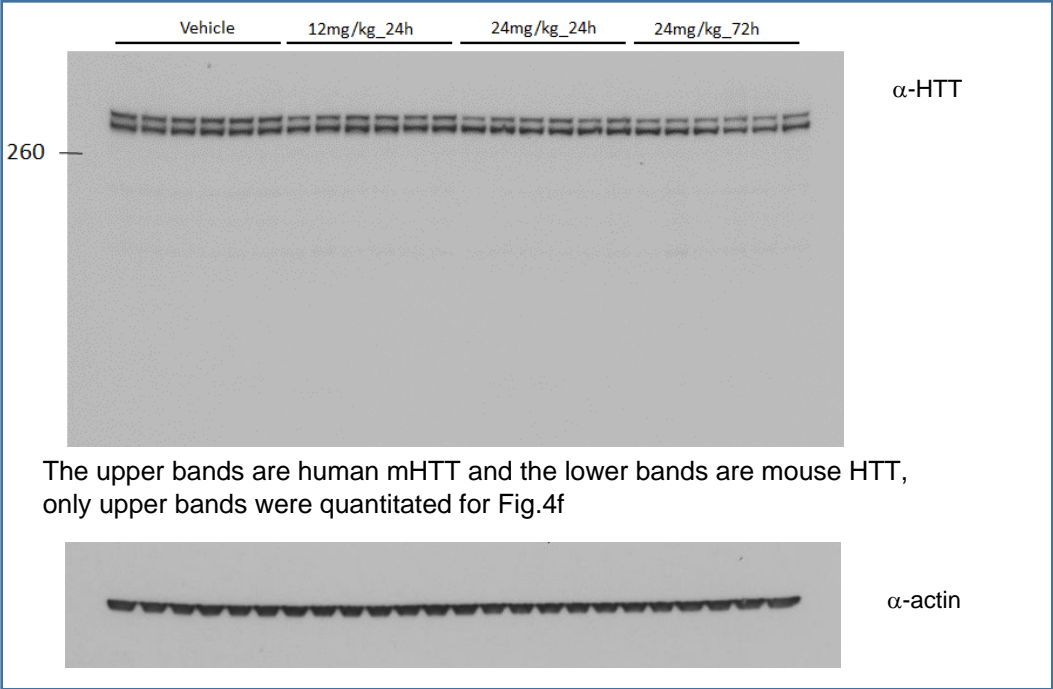

Fig. 4g

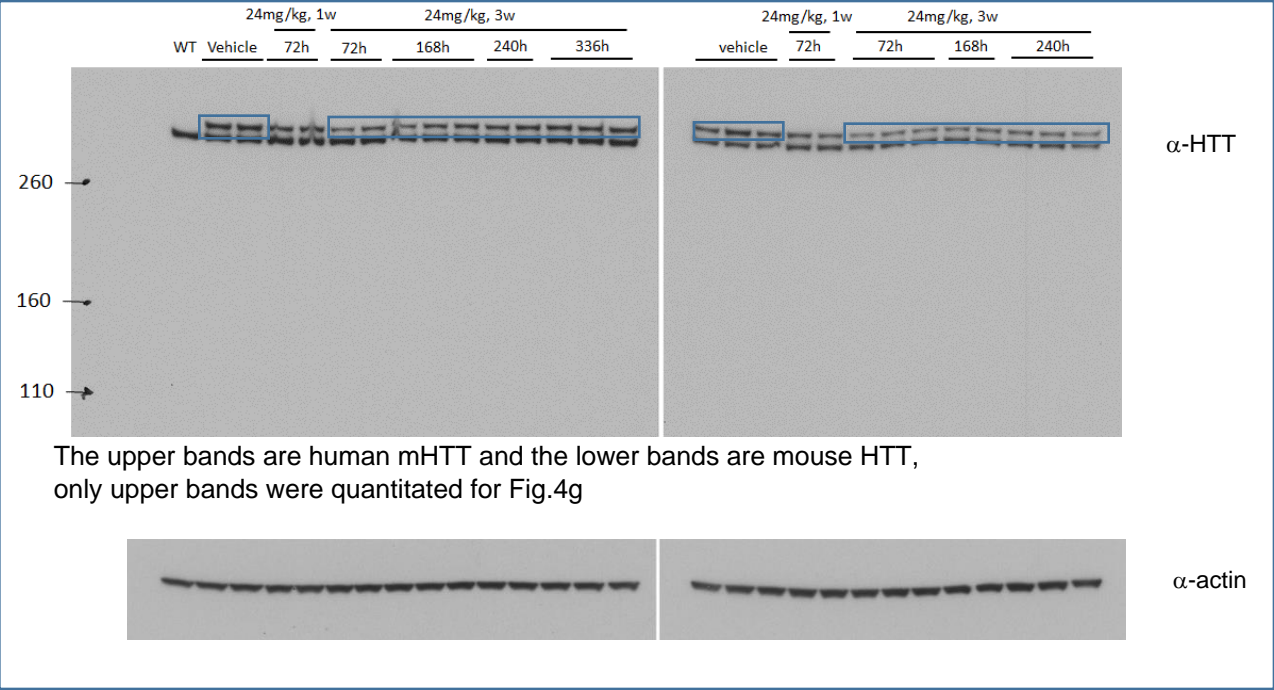

Supplement: Supplementary file 4 — Source Data [file 41467_2022_28653_MOESM4_ESM.zip › 41467_2022_28653_MOESM4_ESM/Gubser Keller et al_Scans of Gels used for Main Figures_Nature Comms_FINAL.pdf]
