# Supplementary material for: An orally available, brain penetrant, small molecule lowers huntingtin levels by enhancing pseudoexon inclusion
Source: Nat Commun. 2022 Mar 3;13:1150. doi: 10.1038/s41467-022-28653-6 (PMC8894458; doi:10.1038/s41467-022-28653-6)
Supplement: Supplementary file 2 — Peer Review File [file 41467_2022_28653_MOESM2_ESM.docx]

**Editorial Note:** This manuscript has been previously reviewed at another journal that is not operating a transparent peer review scheme. The manuscript was considered suitable for publication without further review at *Nature Communications*.
